# Targeting a chemo-induced adaptive signaling circuit confers therapeutic vulnerabilities in pancreatic cancer

**DOI:** 10.1038/s41421-024-00720-w

**Published:** 2024-10-29

**Authors:** Yohei Saito, Yi Xiao, Jun Yao, Yunhai Li, Wendao Liu, Arseniy E. Yuzhalin, Yueh-Ming Shyu, Hongzhong Li, Xiangliang Yuan, Ping Li, Qingling Zhang, Ziyi Li, Yongkun Wei, Xuedong Yin, Jun Zhao, Seyed M. Kariminia, Yao-Chung Wu, Jinyang Wang, Jun Yang, Weiya Xia, Yutong Sun, Eek-hoon Jho, Paul J. Chiao, Rosa F. Hwang, Haoqiang Ying, Huamin Wang, Zhongming Zhao, Anirban Maitra, Mien-Chie Hung, Ronald A. DePinho, Dihua Yu

**Affiliations:** 1 Department of Molecular and Cellular Oncology, The University of Texas MD Anderson Cancer Center, Houston, TX USA; 2 MD Anderson Cancer Center UTHealth Graduate School of Biomedical Sciences, Houston, TX USA; 3 Center for Precision Health, McWilliams School of Biomedical Informatics, The University of Texas Health Science Center at Houston, Houston, TX USA; 4 Department of Biostatistics, The University of Texas MD Anderson Cancer Center, Houston, TX USA; 5 Department of Anatomical Pathology, The University of Texas MD Anderson Cancer Center, Houston, TX USA; 6 Department of Life Science, University of Seoul, Seoul, Korea; 7 Department of Breast Surgical Oncology, The University of Texas MD Anderson Cancer Center, Houston, TX USA; 8 Department of Translational Molecular Pathology, The University of Texas MD Anderson Cancer Center, Houston, TX USA; 9 Human Genetics Center, School of Public Health, The University of Texas Health Science Center at Houston, Houston, TX USA; 10 Departments of Cancer Biology, The University of Texas MD Anderson Cancer Center, Houston, TX USA

**Keywords:** Cancer therapeutic resistance, Cancer microenvironment, Pancreatic cancer

## Abstract

Advanced pancreatic ductal adenocarcinomas (PDACs) respond poorly to all therapies, including the first-line treatment, chemotherapy, the latest immunotherapies, and KRAS-targeting therapies. Despite an enormous effort to improve therapeutic efficacy in late-stage PDAC patients, effective treatment modalities remain an unmet medical challenge. To change the status quo, we explored the key signaling networks underlying the universally poor response of PDAC to therapy. Here, we report a previously unknown chemo-induced symbiotic signaling circuit that adaptively confers chemoresistance in patients and mice with advanced PDAC. By integrating single-cell transcriptomic data from PDAC mouse models and clinical pathological information from PDAC patients, we identified Yap1 in cancer cells and Cox2 in stromal fibroblasts as two key nodes in this signaling circuit. Co-targeting Yap1 in cancer cells and Cox2 in stroma sensitized PDAC to Gemcitabine treatment and dramatically prolonged survival of mice bearing late-stage PDAC, whereas simultaneously inhibiting Yap1 and Cox2 only in cancer cells was ineffective. Mechanistically, chemotherapy triggers non-canonical *Yap1* activation by nemo-like kinase in 14-3-3ζ-overexpressing PDAC cells and increases secretion of CXCL2/5, which bind to CXCR2 on fibroblasts to induce Cox2 and PGE2 expression, which reciprocally facilitate PDAC cell survival. Finally, analyses of PDAC patient data revealed that patients who received Statins, which inhibit Yap1 signaling, and Cox2 inhibitors (including Aspirin) while receiving Gemcitabine displayed markedly prolonged survival compared to others. The robust anti-tumor efficacy of Statins and Aspirin, which co-target the chemo-induced adaptive circuit in the tumor cells and stroma, signifies a unique therapeutic strategy for PDAC.

## Introduction

Pancreatic ductal adenocarcinoma (PDAC) is an aggressive and lethal type of cancer^1^. More than 80% of PDAC patients are diagnosed with unresectable late-stage cancers^2^, which respond poorly to available treatments. PDACs adapt to and resist various therapies, even the latest immunotherapies^3^ and KRAS-targeting therapies^4^. Understanding the fundamental mechanisms underlying the universally poor response of PDAC to therapy remains an unmet challenge.

Currently, nearly all patients with advanced PDAC receive conventional chemotherapies^2^. Gemcitabine (Gem)-based therapies and FOLFIRINOX (5-fluorouracil, oxaliplatin, leucovorin, and irinotecan) are the first-line treatments for advanced PDAC. FOLFIRINOX extends the median survival of PDAC patients, but most patients, especially seniors, cannot tolerate this highly toxic treatment^5^. Gem has been a standard-of-care agent for advanced PDAC for decades, and approximately 50% of patients with PDAC are treated with Gem-based regimens. Although Gem monotherapy and Gem-based combinatorial therapies offer a modest survival benefit, resistance to Gem occurs in most PDACs. In recent decades, only two of more than 20 phase II/III clinical trials designed to improve Gem efficacy in late-stage PDAC showed even minimal improvement over Gem-treatment alone^6,7^. These failures demonstrate the remarkable resilience of PDACs under cytotoxic stress. Additionally, since Gem-based chemotherapy was included in all treatments, Gem may induce adaptive responses that empower PDAC resistance to other treatments. Thus, comprehending how PDACs adapt to chemotherapy is crucial.

The PDAC stromal microenvironment is a fundamental determinant of its biology and therapeutic response. During tumorigenesis, activated fibroblasts from pancreatic stellate cells (PSCs) and bone marrow-derived fibroblasts can make up to 90% of PDAC tissue and produce a hypovascular tumor microenvironment (TME) with high interstitial pressure protecting cancer cells from exposure to drugs^2^. Moreover, PDAC cells co-evolve with the stromal TME that supports tumor immune evasion, metastasis, and resistance to therapy-induced cytotoxic stress. Recently, single-nucleus and single-cell RNA sequencing (snRNA-seq and scRNA-seq) analyses^8,9^ of PDAC tissues from patients after neoadjuvant therapy uncovered the broad impact of chemotherapy on PDAC cells and stroma that may orchestrate the adaptive responses to treatment. However, key signals driving PDAC adaptative responses to chemotherapy in the stromal TME remain unclear. Here, we strive to elucidate the key signaling networks of the adaptive responses to chemotherapy and identify targetable vulnerabilities to facilitate the development of effective PDAC treatment.

## Results

### 14-3-3ζ-overexpressing PDAC enlists the TME to confer Gem resistance

**Fig. 1:**
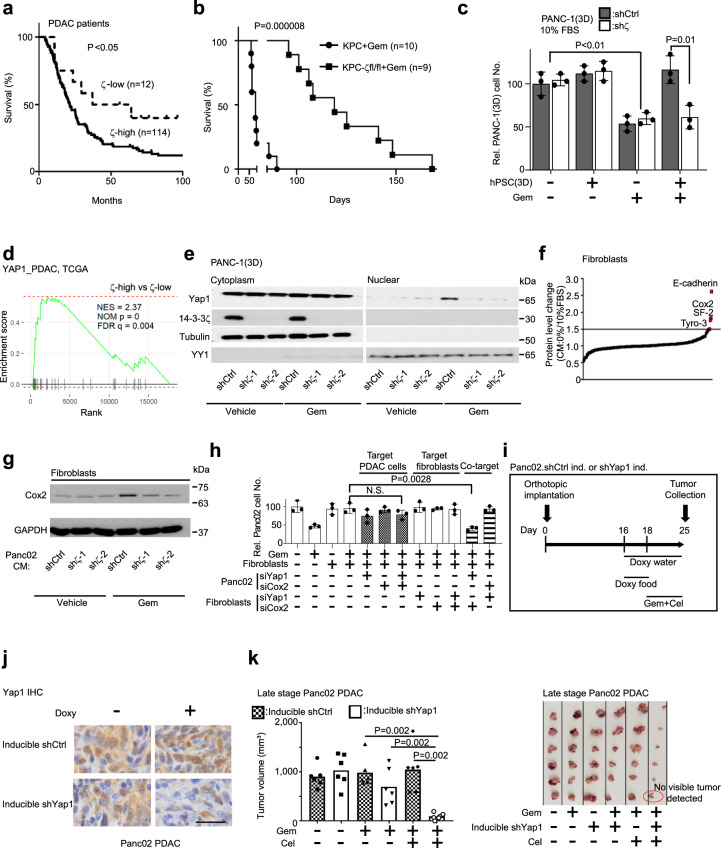
14-3-3ζ-induced Yap1 activation in PDAC cells and Cox2 in fibroblasts confer adaptive Gem resistance that is inhibited by co-targeting Yap1 and Cox2. **a** Kaplan–Meier survival analysis of patients with 14-3-3ζ-high (*n* = 114) and -low expressing (*n* = 12) PDACs (log-rank test). **b** Kaplan–Meier survival analysis of KPC (*n* = 10) and KPC*-ζ*^*fl/fl*^ (*n* = 9) mice treated with Gem (Log-Rank test). **c** Relative cell number of PANC-1.shCtrl/sh*ζ* cells 3D-cultured in the lower chambers of a Transwell unit with or without 3D-cultured hPSCs in the upper chambers of a Transwell unit treated with Gem (20 nM) for 72 h (mean ± SD, *t*-test, *n* = 3 biological repeats). **d** Gene set enrichment analysis (GSEA) of Yap1 signature in 14-3-3ζ-high vs 14-3-3ζ-low human PDACs in the TCGA dataset. **e** Western blotting (WB) analyses of cytoplasmic and nuclear Yap1, 14-3-3ζ, tubulin (a cytoplasmic protein marker, sample processing controls), and YY1 (a nuclear protein marker, sample processing controls) in 3D-cultured PACN-1.shCtrl vs PACN-1.sh*ζ* cells that were treated with Gem (20 nM) or vehicle for 3 h. Representative data of two independent repeats. **f** RPPA analysis of NIH3T3 cells treated with CM collected from Panc02 cells cultured in 10% or 0% FBS medium. **g** WB analysis of Cox2 and GAPDH (sample processing controls) in NIH3T3 cells treated with CM from Panc02.shCtrl or sh*ζ* cells treated with or without Gem (20 nM, 72 h). Representative data of two independent repeats. **h** Relative cell number of Panc02 cells under indicated modifications and conditions; Panc02-GFP cells and NIH3T3 cells were transfected with control or indicated siRNAs respectively, then co-cultured and treated with Gem (8.5 nM) or vehicle. Panc02:NIH3T3 = 1:9 (mean ± SD, *t*-test, *n* = 3 biological repeats). **i** Schematics of in vivo experiment in Fig. 1j, k. Panc02.shCtrl ind. are Panc02.doxy-inducible shCtrl cells, Panc02.sh*Yap1* ind. are Panc02.doxy-inducible sh*Yap1* cells. **j** IHC staining of Yap1 in PDACs from intrapancreatic injection of Panc02.doxy-inducible shCtrl and Panc02.doxy-inducible sh*Yap1* cells. scale bar: 25 µm. **k** Left: Quantification of tumor volumes of doxy-treated mice bearing Panc02.doxy-inducible shCtrl or Panc02.doxy-inducible sh*Yap1* tumors 1 week after the indicated treatments (mean, Mann–Whitney test). Right: Images of treated Panc02 tumors.

As chemotherapy is the standard first-line treatment for most patients with advanced PDACs, patient clinical outcome is a surrogate indicator of their chemotherapy response. To uncover potential factors in PDAC adaptive response to chemotherapy, we analyzed the PDAC transcriptome and proteome in The Cancer Genome Atlas (TCGA)^10^ for genes highly expressed in PDAC and conferred an unfavorable clinical outcome. Among the 668 genes whose expression was significantly associated with unfavorable clinical outcomes, *YWHAZ* (*14-3-3ζ*) was a top candidate highly expressed in PDACs (Supplementary Fig. S1a). Our immunohistochemistry (IHC) analyses of a PDAC patient cohort (most of whom received chemotherapy) from MD Anderson Cancer Center (MDACC) confirmed 14-3-3ζ overexpression (14-3-3ζ+++) in 90% of PDACs with high numbers of Ki-67-positive PDAC cells (Supplementary Fig. S1b, c). These patients with 14-3-3ζ+++ PDACs exhibited much worse survival outcomes than those 10% of patients with 14-3-3ζ low-expression PDACs (Fig. 1a).

14-3-3ζ, an evolutionally conserved adapter protein, regulates many signaling pathways involved in numerous biological functions^11^. To explore 14-3-3ζ function in PDAC, we crossed *14-3-3ζ* gene floxed mice (*ζ*^*fl/fl*^)^12^ with *P48-Cre*;*LSL-Kras*^*G12D*^*;Trp53*^*fl/fl*^ (KPC) mice to generate the pancreas-specific *14-3-3ζ* knockout (KPC*-ζ*^*fl/fl*^) mice (Supplementary Fig. S1d–f). KPC*-ζ*^*fl/fl*^ and KPC mice showed comparable median survival (Supplementary Fig. S1g), suggesting a limited impact of 14-3-3ζ on PDAC initiation and progression. To investigate 14-3-3ζ function in PDAC chemoresistance, which could cause poor clinical outcomes in PDAC patients (Fig. 1a), KPC and KPC*-ζ*^*fl/fl*^ mice were treated with Gem starting at 55 days of age when late-stage PDACs had developed. KPC*-ζ*^*fl/fl*^ mice were exceptionally sensitive to Gem, which dramatically increased survival even though the treatment started at a late stage (Fig. 1b), whereas KPC mice showed no survival benefit from Gem-treatment (Supplementary Fig. S1h), similar to a previous report^13^. To study 14-3-3ζ-mediated Gem resistance, PDAC cell lines were generated from PDAC tumors of KPC and KPC*-ζ*^*fl/fl*^ mice (Supplementary Fig. S1i) and treated with Gem in vitro. Intriguingly, KPC and KPC*-ζ*^*fl/fl*^ cells showed similar cell growth with or without Gem-treatment (Supplementary Fig. S1j). We further tested another three PDAC cell lines that harbor three top driver mutations (*Kras/p53/Smad4*) in human PDAC, namely human Capan-1 (Kras^G12V^), human PANC-1 (Kras^G12D^ and P53^R273H^), and mouse Panc02 (Smad4)^14,15^. *14-3-3ζ* shRNA (sh*ζ*) knockdown in these PDAC cell lines did not alter cell growth compared to control shRNA (shCtrl)-transduced cells under Gem-treatment in two-dimensional (2D) or 3D culture simulating tumor tissue-like structures^16^ (Supplementary Fig. S1k, l). Thus, although 14-3-3ζ was required for Gem resistance in vivo (Fig. 1b), reducing 14-3-3ζ expression did not change PDAC cell growth and response to Gem in vitro, implying that 14-3-3ζ+++ PDAC cells interact with their TME to confer Gem resistance. Since the desmoplastic stroma is the most prominent and unique tissue of the PDAC TME, we explored whether 14-3-3ζ+++ PDAC cells cooperate with PSCs/fibroblasts in the adaptive response to Gem. PANC-1.shCtrl and sh*ζ* cells treated by vehicle or Gem were 3D co-cultured with human PSCs (hPSCs). Under Gem-treatment, PANC-1.shCtrl and sh*ζ* cells had decreased proliferation in single culture without hPSCs, whereas PANC-1.shCtrl cancer cells co-cultured with hPSCs had Gem resistance, which was abolished in PANC-1.sh*ζ* cells (Fig. 1c). Similar data were found using mouse Panc02 cells co-cultured with mouse fibroblasts (NIH3T3) (Supplementary Fig. S2a). To explore whether the Gem-induced symbiotic adaptation between 14-3-3ζ+++ PDAC cells and PSCs/fibroblasts is a general adaptive response to stress, we co-cultured Capan-1.sh*ζ* and shCtrl cells with hPSCs and Panc02.sh*ζ* and shCtrl cells with primary mouse PSCs (mPSCs) under low-serum stress conditions (0%–0.5% fetal bovine serum (FBS)), simulating nutrient deficiency in PDAC TME^17^. No difference in cell proliferation was observed between sh*ζ* and shCtrl PDAC cells in 0%–0.5% FBS without PSCs, whereas shCtrl cancer cells showed significantly increased growth relative to sh*ζ* cells when co-cultured with PSCs in 0%–0.5% FBS (Supplementary Fig. S2b, c).

### Gem induces Yap1 activation in 14-3-3ζ+++ cancer cells and Cox2 expression in PSCs/fibroblasts

Our findings prompted us to explore 14-3-3ζ-mediated druggable targets in PDAC cells and/or PSCs/fibroblasts to improve Gem efficacy since 14-3-3ζ is currently undruggable. Our pathway enrichment analyses of 14-3-3ζ+++ vs 14-3-3ζ low-expressing (14-3-3ζ+) human PDACs from the TCGA and ICGC datasets revealed significantly upregulated *Yap1* signature in 14-3-3ζ+++ vs 14-3-3ζ+ PDACs (Fig. 1d; Supplementary Fig. S3a–c). Interestingly, our analyses of a PDAC snRNA-seq dataset^18^ revealed that 44 of 57 (77%) known Yap1 target genes^19^ were induced in neoadjuvant chemotherapy-treated PDACs (Supplementary Fig. S3d). Additionally, Gem-treatment induced nuclear Yap1 accumulation in 14-3-3ζ+++ PDAC.shCtrl cells (PANC-1 in 3D culture and Panc02) but did not in 14-3-3ζ+ PDAC.sh*ζ* cells (Fig. 1e; Supplementary Fig. S3e). Gem-induced Yap1 nuclear translocation was restored in 14-3-3ζ+ PANC-1.sh*ζ* cells by re-expressing 14-3-3ζ (Supplementary Fig. S3f), indicating that 14-3-3ζ is essential for Gem-induced Yap1 activation in PDAC cells. To explore the Gem-driven genome-wide regulatory program in 14-3-3ζ+++ PDAC cells, RNA-seq was performed using Gem-treated, 3D-cultured KPC mT3 cell line, which was established from a PDAC lesion in *Pdx1-Cre*;*LSL-Kras*^*G12D*^;*LSL-Trp53*^*R172H*^ mice^20^. Gem-treatment of KPC mT3 cells induced expression of many Yap1 target genes (Supplementary Fig. S4a–c), including CTGF/CCN2 and CYR61/CCN1, with critical functions in promoting desmoplastic reaction in TME and PDAC drug resistance^21,22^. We further confirmed that Gem-induced Yap1 nuclear accumulation in the 3D-cultured KPC mT3 cells (Supplementary Fig. S4d). These data suggest that Gem induces Yap1 signaling activation as part of the adaptive response that may contribute to 14-3-3ζ-mediated Gem resistance in PDACs. Moreover, under serum-free conditions, 14-3-3ζ+++ PDAC cells also had increased nuclear Yap1 compared to 14-3-3ζ-low cells, despite similar total Yap1 levels in these cells (Supplementary Fig. S5a, b). Yap1 can be targeted by several clinically applicable drugs, such as Verteporfin (VP) and Protoporphyrin IX (PPIX)^23^. Additionally, Statins, a class of lipid-lowering medications used to prevent cardiac disease, inhibit Yap1 nuclear localization and transcriptional responses in human cancer cells^24,25^.

Since 14-3-3ζ+++ PDAC cells cooperate with PSCs/fibroblasts for adaptation to stresses, we examined the impact of stressed PDAC cells on PSCs/fibroblasts by performing reverse phase protein array (RPPA) on fibroblasts cultured in conditioned medium (CM) from Panc02.shCtrl cells grown in 0% FBS vs 10% FBS. Of the four most up-regulated proteins (E-cadherin, SF-2, Tyro-3, and Cox2) (Fig. 1f), Cox2, which is a key mediator of inflammatory pathways, was consistently upregulated in PSCs/fibroblasts when cultured in CM from 14-3-3ζ+++ shCtrl (Panc02.shCtrl, Capan-1.shCtrl, and PANC-1.shCtrl) cells, but not corresponding 14-3-3ζ + sh*ζ* cells, grown in 0% FBS or treated with Gem (Fig. 1g; Supplementary Fig. S6a, b). Also, CM from Gem-treated and 14-3-3ζ expression-rescued PANC1.sh*ζ* cells induced Cox2 expression in hPSCs (Supplementary Fig. S6c). However, neither Gem nor 0% FBS directly induced Cox2 expression in fibroblasts without CM of 14-3-3ζ+++ PDAC cells (Supplementary Fig. S6d). These data suggested that stressed 14-3-3ζ+++ PDAC cells induce Cox2 upregulation in PSCs/fibroblasts and that the induction of Cox2 is part of the adaptive response to Gem-treatment.

### Co-targeting cancer cell Yap1 and stromal Cox2 sensitizes PDAC to Gem-treatment

The above data indicated that Gem induces Yap1 activation in 14-3-3ζ+++ PDAC cells and Cox2 upregulation in PSCs/fibroblasts. If that contributes to Gem resistance in 14-3-3ζ+++ PDACs, targeting Yap1 and/or Cox2 would improve Gem response. Thus, we targeted Yap1 and Cox2 singly or together by siRNA in Panc02 cells and fibroblasts (Supplementary Fig. S6e) that were co-cultured under Gem-treatment (Fig. 1h). Single targeting of Yap1 in Panc02 cancer cells or Cox2 in fibroblasts did not enhance Gem response compared to Gem mono-treatment. Dual-targeting Yap1 and Cox2 solely in Panc02 cells or in fibroblasts had no discernable effect on Gem response, unlike previous reports that dual-targeting Yap1 and Cox2 only in cancer cells enhances chemotherapy efficacy in colon cancer and bladder cancer^26,27^. By contrast, the knockdown of Yap1 in Panc02 cells and of Cox2 in fibroblasts significantly enhanced Gem response (Fig. 1h), indicating that Yap1 in PDAC cells and Cox2 in fibroblasts are essential for 14-3-3ζ+++ PDAC cell adaptive response to Gem-induced stress. To mimic the therapeutic targeting of Yap1 in vivo, Panc02 cells were stably transfected with doxycycline (doxy)-inducible Yap1-targeting shRNA (sh*Yap1* ind) or control shRNA (shCtrl ind) vectors and injected into the pancreas of mice. At 16 days after injection, when Panc02 tumors were readily palpable and produced ascites, which are clinical features of advanced pancreatic cancers^28^, mice were fed with doxy-containing water and food to knock down Yap1, followed by treatment with Cox2 inhibitor Celecoxib (Cel)^29^ and/or Gem starting at day 18 (Fig. 1i, j). Remarkably, co-targeting Yap1 by sh*Yap1* and Cox2 by Cel-treatment converted Gem-resistant late-stage PDACs to highly Gem-sensitive with one PDAC tumor regressed to become undetectable (Fig. 1k). These results demonstrated that co-targeting Yap1 and Cox2 can boost Gem response to achieve high efficacy and that Yap1 in PDAC cells and Cox2 in fibroblasts contribute to Gem resistance.

### 14-3-3ζ-overexpressing PDAC cells cooperate with PSCs/fibroblasts in stress adaptation

The efficacy of co-targeting Yap1 in PDAC cells and Cox2 in fibroblasts in vitro and in late-stage PDACs in vivo (Fig. 1h–k) highlight the codependent functions of Yap1 activation in cancer cells and of Cox2 upregulation in PSCs/fibroblasts in the Gem resistance of 14-3-3ζ+++ PDACs. Thus, we tested whether the adaptive responses to Gem are initiated from PSCs/fibroblasts or 14-3-3ζ+++ PDAC cells. Panc02.shCtrl and Panc02.sh*ζ* cells proliferated similarly (Supplementary Fig. S7a) in fibroblast CM (F-CM) from Gem-treated, or 0% FBS-cultured fibroblasts or mPSCs, indicating that adaptive responses to Gem are not initiated by PSCs/fibroblasts. By contrast, F-CM from fibroblasts or mPSCs cultured in CM from Gem-treated or 0% FBS-cultured 14-3-3ζ+++ Panc02.shCtrl cells dramatically increased Panc02.shCtrl PDAC cell proliferation and Gem-resistance compared to that in CM from Gem-treated or 0% FBS-cultured 14-3-3ζ+ Panc02.sh*ζ* cells (Supplementary Fig. S7b). Similar results were found using 3D-cultured hPSCs and Gem-treated PANC-1 cells (Supplementary Fig. S7c). Reciprocally treating Panc02.sh*ζ* cells with F-CM from fibroblasts activated by Panc02.shCtrl CM and vice versa showed that F-CM educated by Panc02.shCtrl CM (not Panc02.sh*ζ* CM) enhanced Gem resistance to PDAC.sh*ζ* cells (Supplementary Fig. S7d), suggesting a crucial non-cell-autonomous role of 14-3-3ζ in PDAC cells. Taken together, these data suggest that under stress conditions (Gem-treatment or nutrient deficiency), 14-3-3ζ+++ cancer cells initiate adaptive responses by secreting factors to activate PSCs/fibroblasts, which in turn facilitates cancer cell adaptation to stresses.

### Stress-induced Nemo-like kinase (NLK) protein stabilization facilitates Yap1 nuclear accumulation

**Fig. 2:**
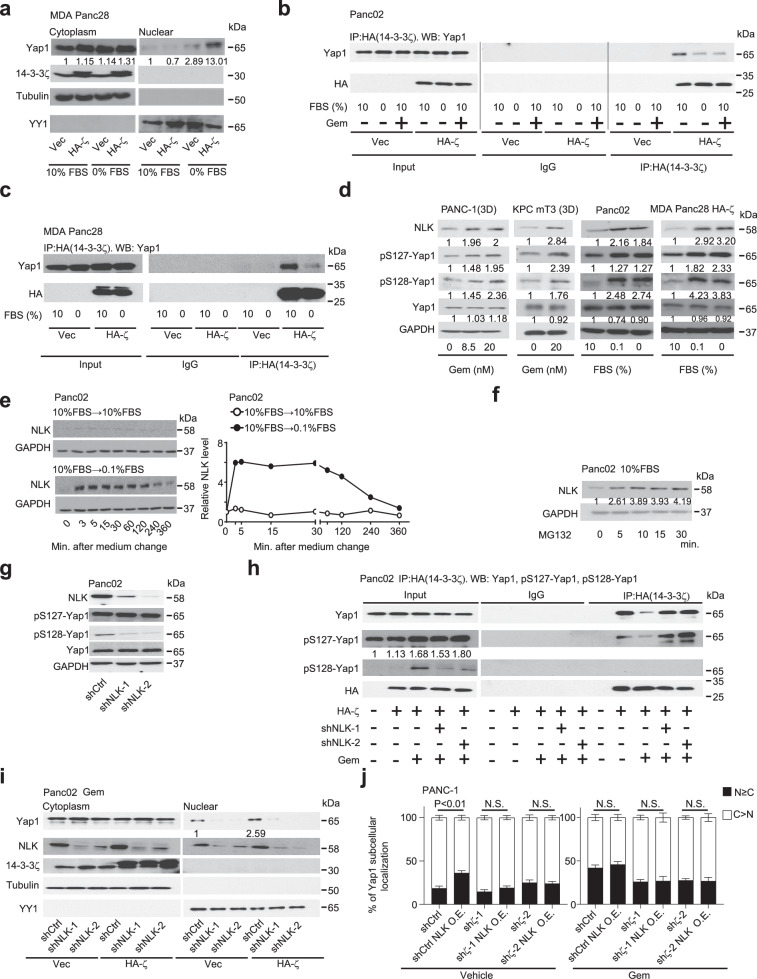
Stress inhibits NLK degradation to increase Yap1 phosphorylation on Ser128 and nuclear translocation in 14-3-3ζ+++ PDAC cells. **a** WB analysis of nuclear and cytoplasmic Yap1, 14-3-3ζ, tubulin (sample processing controls), and YY1 (sample processing controls) in MDA Panc28. Vec cells or MDA Panc28.HA-ζ cells cultured in 10% or 0% FBS medium for 24 h. **b** Immunoprecipitation (IP) of HA-tagged 14-3-3ζ followed by WB of Yap1 and HA-tag in Panc02.Vec or HA-ζ cells cultured in 10% or 0% FBS (2 h), or with Gem-treatment (20 nM, 3 h) in 10% FBS medium. **c** IP of HA-tagged 14-3-3ζ followed by WB of Yap1 and HA-tag in MDA Panc28.Vec or HA-ζ cells cultured in 10% or 0% FBS medium (24 h). **d** WB analysis of NLK, pS127-Yap1, pS128-Yap1, total Yap1, and GAPDH (sample processing controls) in 3D-cultured PANC-1 cells and KPC mT3 cells treated with indicated dosages of Gem vs vehicle for 30 min (PANC-1 cells) or 45 min (KPC mT3 cells), and in Panc02 cells and MDA Panc28.HA-ζ cells cultured in 10%, 0.1%, or 0% FBS for 30 min (Panc02 cells) or 2 h (MDA Panc28.HA-ζ cells). **e** WB analyses of NLK and GAPDH (sample processing controls) in Panc02 cells cultured in 10% FBS then switched to 10% or 0.1% FBS for the indicated times (0–360 min). Right: Quantification of NLK protein levels. The starting point (0 min) data were defined as 1. **f** WB analyses of NLK and GAPDH (sample processing controls) in Panc02 cells cultured in 10% FBS added with MG132 (20 μM) for the indicated times. **g** WB analyses of NLK, pS127-Yap1, pS128-Yap1, total Yap1, and GAPDH (sample processing controls) in Panc02.shCtrl and two sublines of Panc02.sh*NLK* cells cultured in 0.1% FBS for 30 min. **h** IP of HA-tagged 14-3-3ζ followed by WB of Yap1, pS127-Yap1, pS128-Yap1, and HA-tag in Vec or HA-ζ overexpressing Panc02.shCtrl or sh*NLK* cells with Gem-treatment (20 nM, 3 h) in 10% FBS medium. **i** WB analyses of cytoplasmic and nuclear Yap1, NLK, 14-3-3ζ, tubulin (sample processing controls), and YY1 (sample processing controls) in Vec or HA-ζ overexpressing Panc02.shCtrl and Panc02.sh*NLK* sublines with Gem-treatment (20 nM, 3 h) in 10% FBS medium. **j** IF staining of Yap1 in Vec or NLK overexpressing PANC-1.shCtrl or PANC-1.sh*ζ* cells with or without Gem (20 nM) for 3 h (mean ± SEM, two-way ANOVA, 10 representative pictures for each group). All data are representative of at least two independent repeats.

Our above data demonstrate that Gem-treatment and low-nutrient induce Yap1 nuclear accumulation and signaling in 14-3-3ζ+++ PDAC cells to activate PSCs/fibroblasts. However, 14-3-3ζ is known to retain Yap1 in the cytoplasm by binding to phospho-Ser127-Yap1 (pS127-Yap1) upon canonical hippo-pathway activation^30^ or under serum-free cell culture^31,32^. Since many of these previous studies were done in non-transformed cells under serum-free conditions^31,32^, we wondered whether 14-3-3ζ functions differently in non-transformed pancreatic epithelial cells vs PDAC cells regarding stress-induced Yap1 activation. Indeed, overexpressing HA-tagged 14-3-3ζ in non-transformed human pancreas duct epithelial cells (HPDE/E6E7) reduced Yap1 nuclear localization under serum-free conditions (Supplementary Fig. S8a, b). By contrast, when HA-tagged 14-3-3ζ was overexpressed in MDA-Panc28 PDAC cells, which have endogenous 14-3-3ζ levels similar to HPDE/E6E7 cells (Supplementary Fig. S8c, d), low-serum stress strongly increased nuclear Yap1 accumulation compared to control vector (Vec)-transfected MDA-Panc28 cells (Fig. 2a), echoing data from 14-3-3ζ-knockdown PDAC cell lines (Fig. 1e; Supplementary Figs. S3e, 5a). Thus, 14-3-3ζ+++ PDAC cells regulate Yap1 subcellular localization differently from non-transformed pancreas epithelial cells. Next, we examined whether stress alters the interaction between Yap1 and 14-3-3ζ in PDAC cells. Yap1 binding to 14-3-3ζ was reduced under Gem-treatment or 0% FBS culture in HA-ζ expressing mouse (Panc02.HA-ζ) and human (MDA-Panc28.HA-ζ) PDAC cells (Fig. 2b, c). Yap1 nuclear accumulation and activation following the decreased 14-3-3ζ binding usually results from dephosphorylation of pSer127-Yap1, which is a critical 14-3-3ζ binding site^32,33^. However, pS127-Yap1 levels were increased in 14-3-3ζ+++ PDAC cells by stressors, despite the reduced binding of 14-3-3ζ and Yap1 (Fig. 2d), suggesting that pS127-Yap1 binding to 14-3-3ζ in PDAC cells was inhibited under stress conditions. NLK-mediated Yap1 phosphorylation at Ser128 (pS128-Yap1) has been reported to inhibit 14-3-3 binding and increase Yap1 nuclear localization^34,35^. Importantly, pS128-Yap1 was shown to override Ser127 phosphorylation and pS127-Yap1-mediated Yap1 cytoplasmic sequestration^34^. Indeed, NLK expression and pS128-Yap1 were increased in PDAC cells under stresses (Fig. 2d). The stress-induced NLK upregulation resulted from increased NLK protein stability, not mRNA upregulation (Fig. 2e, f; Supplementary Fig. S8e). Notably, stress-induced NLK high expression was detected in Panc02.Vec and Panc02.HA-ζ PDAC cells as well as in Panc02.shCtrl vs sh*ζ* cells (Supplementary Fig. S8f), suggesting that stress-induced NLK stabilization is independent of 14-3-3ζ expression.

To examine the function of NLK in Gem-induced Yap1 nuclear accumulation and stress resistance, NLK was knocked down in Panc02 cells (Panc02.shNLK), which resulted in abolished stress resistance triggered by F-CM under stresses (Supplementary Fig. S8g). By contrast, CM from Panc02.shNLK didn’t show a significant cell-autonomous effect on PDAC cell growth (Supplementary Fig. 8h). Importantly, pS128-Yap1 level, but not pS127-Yap1 level, were dramatically reduced in Panc02.sh*NLK* cells (Fig. 2g), indicating that stress-induced NLK upregulation increases S128, not S127, phosphorylation, although stress enhanced phosphorylation of S127 and S128 (Fig. 2d). Additionally, increased 14-3-3ζ-Yap1 binding was detected in sh*NLK* cells compared to that of the shCtrl cells under Gem stress (Fig. 2h), leading to reduced Yap1 nuclear localization (Fig. 2i). Furthermore, the reduced pS127-Yap1 binding with 14-3-3ζ under Gem stress was restored by NLK knockdown, whereas pS128-Yap1 did not show an interaction with 14-3-3ζ (Fig. 2h), implying that pS128 may prohibit Yap1 binding to 14-3-3ζ regardless of pS127 status.

To investigate the impact of S127 and S128 phosphorylation status on Yap1 subcellular localization under stress conditions, Flag-tagged non-phosphorylable mutants S127A-Yap1 and S128A-Yap1, phospho-mimetic mutants S127D-Yap1 and S128D-Yap1, and wide-type (WT) Yap1 were introduced into MDA-Panc28.HA-ζ cells, respectively. The resulting PDAC cell sublines were treated with vehicle or Gem and evaluated for Yap1 subcellular localization by immunofluorescence (IF) staining of Flag-tagged Yap1 (Supplementary Fig. S8i). Consistent with previous reports^33,36^, Flag-Yap1-S127A mutants were mostly detected in the nucleus with or without Gem treatment, whereas Flag-Yap1-S127D phospho-mimetic mutants were mostly retained in the cytosol with modestly increased nucleus translocation in response to Gem-treatment, much less than the extent of WT Yap1 (Supplementary Fig. S8i). On the other hand, Flag-Yap1-S128A non-phosphorylable mutants failed to significantly increase translocation into the nucleus by Gem-treatment, yet Flag-Yap1-S128D phospho-mimetic mutants were mostly detected in the nucleus even without Gem stress (Supplementary Fig. S8i). Together, these data indicate that S127 phosphorylation sequesters Yap1 in the cytosol by its strong binding with 14-3-3ζ, which may be partially overridden by Gem-induced S128 phosphorylation, and S128 phosphorylation is required for Gem-induced Yap1 nuclear translocation likely by overriding the suppressive effect of S127 phosphorylation.

To examine whether NLK, by inducing pS128-Yap1, is sufficient in driving Yap1 nuclear accumulation under stress, we exogenously expressed NLK in NLK-low expressing PANC-1.shCtrl and PANC-1.sh*ζ* PDAC cells (Supplementary Fig. S8j). Indeed, increased Yap1 nuclear localizations were detected in NLK-high-expressing PANC-1.shCtrl (14-3-3ζ+++) cells even without Gem-treatment, indicating Yap1 is constantly activated by overexpression of NLK in 14-3-3ζ+++ cells (Fig. 2j, left). But Yap1 nuclear translocations in *NLK*-transduced PANC-1.shζ (14-3-3ζ+) cells remained low even under Gem-treatment (Fig. 2j, right), indicating that NLK and 14-3-3ζ are required for stress-induced Yap1 activation. Functionally, F-CM from hPSC cultured in CM from NLK-high sh*ζ* cells didn’t rescue the growth inhibition of Gem on PANC-1.sh*ζ* cells (Supplementary Fig. S8k). Collectively, in stressed 14-3-3ζ+++ PDAC cells, stabilized NLK can increase pS128-Yap1, preventing Yap1 binding with 14-3-3ζ, thus facilitating Yap1 nuclear translocation and activation, and ultimately inducing the secretion of factors that activate PSCs/fibroblasts.

### Stressed 14-3-3ζ+++ PDAC cells induce CXCL2/5 via Yap1

**Fig. 3:**
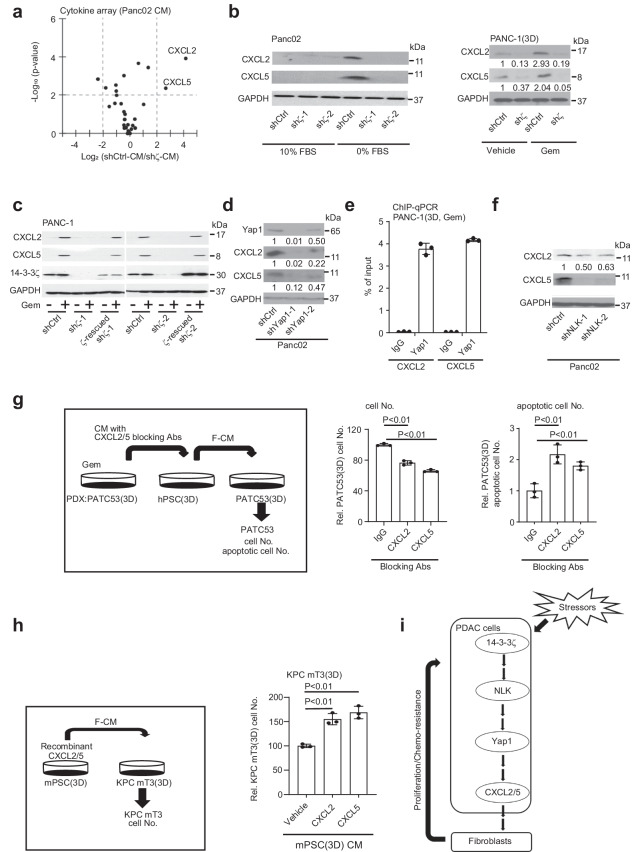
14-3-3ζ overexpressing PDAC cells increases CXCL2/5 via Yap1 in response to stresses. **a** Cytokine array analysis of CM from Panc02.shCtrl vs Panc02.sh*ζ* cells in 0% FBS culture for 72 h. **b** Left: WB analysis of CXCL2, CXCL5, and GAPDH (sample processing controls) expression in Panc02.shCtrl and Panc02.sh*ζ* cells cultured in 10% FBS or 0% FBS medium for 48 h. Right: WB analysis of CXCL2, CXCL5, and GAPDH (sample processing controls) in 3D cultured PANC-1.shCtrl and PANC-1.sh*ζ* cells treated with Gem (20 nM) vs vehicle for 48 h. **c** WB analysis of CXCL2, CXCL5, 14-3-3ζ, and GAPDH (sample processing controls) in PANC-1.shCtrl and PANC-1.shζ, and 14-3-3ζ-overexpressing PANC-1.sh*ζ* cells treated with Gem (20 nM) vs vehicle for 48 h. **d** WB analyses of Yap1, CXCL2, CXCL5, and GAPDH (sample processing controls) expression in Panc02.shCtrl and Panc02.sh*Yap1* cells in 0% FBS culture for 24 h. Representative data of two independent repeats. **e** ChIP-qPCR assays of Yap1 binding to *CXCL2/5* promoter region in 3D-cultured PANC-1 cells with 24 h of Gem (20 nM) treatment (mean ± SD, *t*-test, *n* = 3 biological repeats). **f** WB analysis of CXCL2, CXCL5, and GAPDH (sample processing controls) in Panc02.shCtrl and Panc02.sh*NLK* sublines cultured in 0.1% FBS for 24 h. Representative data of two independent repeats. **g** Schematics (left) and relative numbers of proliferating (middle) and apoptotic (right) cells from 3D-cultured PATC53 cells treated with CM from 3D-cultured hPSCs that were activated by adding CM from 8.5 nM Gem-treated 3D-cultured PATC53 cells plus CXCL2 (1 μg/mL) or CXCL5 (3 μg/mL) blocking antibodies for 48 h (mean ± SD, *t*-test, *n* = 3 biological repeats). **h** Schematics and relative cell number of 3D-cultured KPC mT3 cells treated with CM from 3D-cultured mPSCs added with vehicle, recombinant CXCL2 (0.5 ng/mL), or recombinant CXCL5 (0.1 µg/mL) proteins for 48 h (mean ± SD, *t*-test, *n* = 3 biological repeats). **i** Stress-induced 14-3-3ζ-Yap1-CXCL2/5 pathway in PDAC cells activates fibroblasts, which turns on the adaptive response that enables PDAC cells to survive under stress conditions.

To identify 14-3-3ζ+++ cancer cell-secreted factors that activate PSCs/fibroblasts, CM from Panc02.shCtrl and Panc02.sh*ζ* cells (0% FBS culture) were profiled for cytokine secretions, revealing elevated levels of CXCL2 and CXCL5 in CM from 14-3-3ζ+++ Panc02.shCtrl cells (Fig. 3a). Upregulation of CXCL2/5 proteins and mRNAs were confirmed in multiple 14-3-3ζ+++ (PANC-1.shCtrl, Panc02.shCtrl, and Capan-1.shCtrl, and MDA-Panc28.HA-ζ) PDAC cell lines, as well as PANC-1.sh*ζ* cells with rescued 14-3-3*ζ* expression under various stress conditions (Gem-treatment, 0% FBS, or glucose starvation) (Fig. 3b, c; Supplementary Fig. S9a–d). Yap1 knockdown in Capan1 (Capan1.sh*Yap1*) and in Panc02 (Panc02.sh*Yap1*) cells or *Yap1* knockout in iKras (iKras.sg*Yap1*) PDAC cells from an inducible-*Kras*^*G12D*^ transgenic mouse^37^ reduced CXCL2/5 expression (Fig. 3d; Supplementary Fig. S9e). Moreover, Yap1 binds to *CXCL2/5* promoters in Gem-treated, 3D-cultured PANC-1 cells detected by chromatin immunoprecipitation (ChIP)-qPCR assays (Fig. 3e). NLK knockdown also inhibited CXCL2/5 expression (Fig. 3f). Together, our data indicate that CXCL2/5 is induced by stressors in 14-3-3ζ+++ PDAC cells via NLK-Yap1 pathway.

Next, we tested whether PDAC cell-derived CXCL2/5 is critical for symbiotic adaptation to Gem-treatment in human PDACs using PATC53, a PDAC patient-derived xenograft (PDX) model^38^. We collected CM from Gem-treated 3D-cultured PATC53 cells and added with control IgG or CXCL2/5 blocking antibodies for 3D-culture of hPSCs, then harvested F-CM for 3D-culture of PATC53 PDX (Fig. 3g). PATC53 cultured in F-CM with CXCL2/5 blocking antibodies significantly reduced proliferation and increased apoptosis compared to that with control IgG (Fig. 3g). Similarly, knockdown CXCL2/5 in 14-3-3ζ+++ Panc02 cells (Panc02.shCXCL2/5; Supplementary Fig. S9f) blocked fibroblasts-mediated adaptive response to the 0% FBS stress (Supplementary Fig. S9g, h). Conversely, F-CM from mPSCs and fibroblasts with added recombinant CXCL2/5 proteins increased the proliferation of 3D-cultured KPC mT3 and Panc02 cells (Fig. 3h; Supplementary Fig. S9i). Autocrine CXCL2/5 signaling seemed dispensable for PDAC cell adaptive response to stress since Panc02 or KPC mT3 cell growth was unaltered by CXCL2/5 knockdown (sh*CXCL2/5*) or recombinant CXCL2/5 proteins (Supplementary Fig. S9j, k). Thus, stress activates NLK-Yap1-CXCL2/5 signaling in 14-3-3ζ+++ cancer cells to instigate PSCs/fibroblasts which in turn support cancer cell proliferation and survival (Fig. 3i).

### CXCL2/5 activates the mTORC2-Cox2 axis in PSCs/fibroblasts

**Fig. 4:**
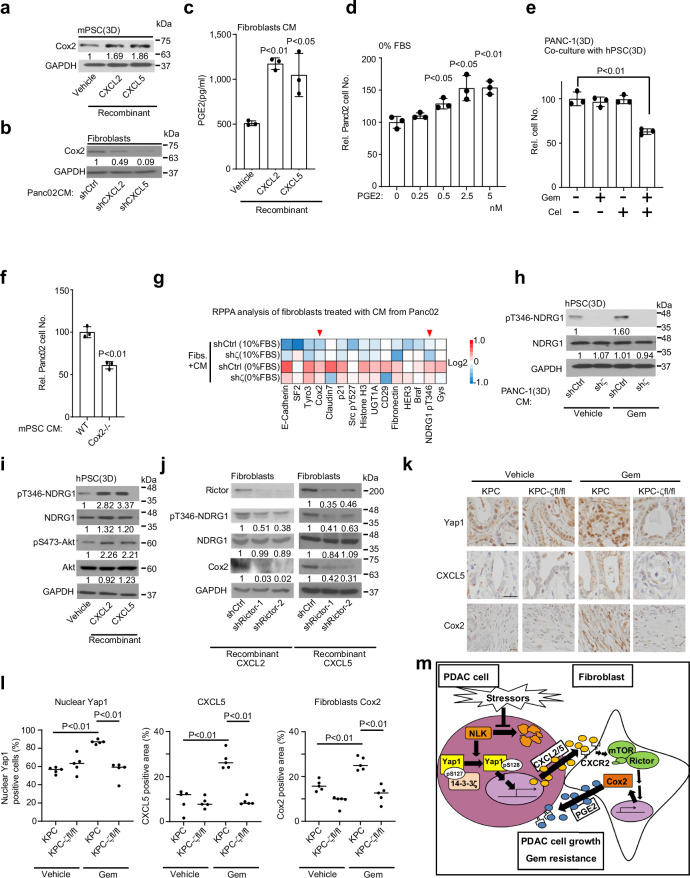
PDAC cell-secreted CXCL2/5 activates the CXCR2-mTORC2-Cox2-PGE2 axis in fibroblasts for stress adaptation of PDAC cells. **a** WB analyses of Cox2 and GAPDH (sample processing controls) in mPSCs (3D culture) incubated with recombinant CXCL2 (0.5 ng/mL) or CXCL5 (0.1 µg/mL) for 24 h. Representative data of two independent repeats. **b** WB analysis of Cox2 and GAPDH (sample processing controls) in NIH3T3 cells treated with CM from Panc02.shCtrl, Panc02.sh*CXCL2*, or Panc02.sh*CXCL5* cells cultured in 0% FBS for 72 h. Representative data of two independent repeats. **c** PGE2 concentration in CM from NIH3T3 cells treated with recombinant CXCL2 (0.5 ng/mL) or CXCL5 (0.1 µg/mL) for 48 h (mean ± SD, *t*-test, *n* = 3 biological repeats). **d** Relative numbers of Panc02 cells treated with PGE2 in 0% FBS for 48 h (mean ± SD, *t*-test, *n* = 3 biological repeats). **e** Relative numbers of PANC-1 cells in upper chambers of Transwell units 3D-cocultured with hPSCs in lower chambers of Transwell units with or without Cel (4 nM) and Gem (20 nM) for 72 h (mean ± SD, *t*-test, *n* = 3 biological repeats). **f** Relative number of Panc02 cells treated with CM from WT mPSCs or *Cox2*^–/–^ mPSCs activated by CM from Panc02 cells in 0% FBS for 48 h (mean ± SD, *t*-test, *n* = 3 biological repeats). **g** RPPA analysis of NIH3T3 cells treated with CM collected from Panc02.shCtrl and Panc02.sh*ζ* cells cultured in 10% or 0% FBS medium. **h** WB analysis of pT346-NDRG1, NDRG1, and GAPDH (sample processing controls) in 3D-cultured hPSCs treated for 24 h with CM collected from 3D-cultured PANC-1.shCtrl and PANC-1.sh*ζ* cells treated with vehicle/Gem (20 nM) for 24 h. Representative data of two independent repeats. **i** WB analysis of pT346-NDRG1, NDRG1, pS473-Akt, Akt, and GAPDH (sample processing controls) expression in 3D-cultured hPSCs treated with recombinant CXCL2 (15 ng/mL) or CXCL5 (25 ng/mL) for 24 h. Representative data of two independent repeats. **j** WB analysis of Rictor, pT346-NDRG1, NDRG1, Cox2, and GAPDH (sample processing controls) protein in NIH3T3.shCtrl and NIH3T3.sh*Rictor* cells treated for 24 h with recombinant CXCL2 (0.5 ng/mL) or recombinant CXCL5 (0.1 µg/mL). Representative data of two independent repeats. **k,** **l** IHC staining of indicated proteins of PDAC tumor tissues from KPC and KPC*-ζ*^*fl/fl*^ mice with indicated treatments (mean ± SD, *t*-test, *n* = 5 biological repeats, scale bar: 20 µm). **m** PDAC cells and fibroblasts together create the adaptive stress response circuit, which is essential for PDAC cell adaptation to stresses, including nutrient deprivation and chemotherapy-induced genotoxicity. Stressors promote the Yap1-CXCL2/5 signaling axis via NLK in 14-3-3ζ-overexpressing PDAC cells, leading to the activation of the CXCR2-mTORC2-Cox2-PGE2 pathway in fibroblasts. Reciprocally, PGE2 from fibroblasts promotes PDAC cell survival and adaptive resistance to Gem.

Since CXCL2/5 derived from stressed 14-3-3ζ+++ PDAC cells is critical for maneuvering PSCs/fibroblasts, and Cox2 induction in PSCs/fibroblasts is part of the adaptive response (Fig. 1g; Supplementary Fig. S6a–d), we investigated whether upregulated CXCL2/5 from stressed 14-3-3ζ+++ cancer cells causally induce Cox2 expression in PSCs/fibroblasts by gain- and loss-of-function studies. Indeed, recombinant CXCL2/5 proteins increased Cox2 protein expression in 3D-cultured mPSCs and fibroblasts (Fig. 4a; Supplementary Fig. S10a), while CM with added CXCL2/5 blocking antibodies or CM from Panc02.sh*CXCL2/5* cells reduced Cox2 expression in 3D-cultured mPSCs and fibroblasts compared to those control CM (Fig. 4b; Supplementary Fig. S10b). Additionally, fibroblasts with knockdown of the CXCR2 receptor for CXCL2/5 growing in Panc02-CM (0% FBS) showed reduced *Cox2* mRNA and protein expression compared to that of control fibroblasts (Supplementary Fig. S10c, d). Thus, 14-3-3ζ+++ PDAC cell-secreted CXCL2/5 upregulates Cox2 expression in fibroblasts via CXCR2. Expectedly, recombinant CXCL2/5 increased Cox2-mediated PGE2 secretion from fibroblasts (Fig. 4c), which was blocked by a Cox2 inhibitor Cel (Supplementary Fig. S10e). PGE2 treatment increased the proliferation of human and mouse PDAC cells in a dose-dependent manner (Fig. 4d; Supplementary Fig. S10f). PDAC cells had reduced proliferation and increased apoptosis when cultured with Cel-treated PSCs/fibroblasts or their F-CM under stress conditions (Fig. 4e; Supplementary Fig. S10g, h), whereas adding Cel directly to Panc02 cells had no noticeable effect (Supplementary Fig. S10i). Similarly, PDAC cell proliferation was reduced when cultured with F-CM of PSCs from *Cox2* knockout (*Cox2*^*–/–*^) mice compared to that from WT mice (Fig. 4f; Supplementary Fig. S10j). Collectively, these data indicated that Cox2 upregulation in PSCs/fibroblasts is a key symbiotic signal of adaptive response to stress and provided mechanistic insight underlying the remarkable efficacy of Gem treatment by co-targeting Cox2 in PSCs/fibroblasts and Yap1 in PDAC cells (Fig. 1h–k).

To explore how 14-3-3ζ+++ cancer cell-secreted CXCL2/5 induce Cox2 expression in PSCs/fibroblasts, we profiled fibroblasts cultured in CM (10% FBS or 0% FBS) of Panc02.shCtrl cells or Panc02.sh*ζ* cells by RPPA. Among the top 15 upregulated proteins of fibroblasts in CM from Panc02.shCtrl cells cultured in 0%FBS, phospho-threonine-346 of NDRG1 (pT346-NDRG1), a well-known mTROC2 activation marker^39^, was the only phospho-protein, along with Cox2 protein, that was increased in fibroblasts activated by stressed Panc02.shCtrl cells vs Panc02.sh*ζ* cells (Fig. 4g). The data were validated in 3D-cultured hPSCs with CM from vehicle- or Gem-treated PANC-1.shCtrl vs sh*ζ* cells (Fig. 4h). pT346-NDRG1 along with another mTORC2 activation marker, phospho-serine-473 of Akt^40^, were also increased in 3D-cultured hPSCs by recombinant human CXCL2/5 (Fig. 4i), whereas fibroblasts cultured in CM from Panc02.sh*CXCL2/5* cells or with CXCR2 knockdown decreased pT346-NDRG1 compared to controls (Supplementary Fig. S10k, l). Knockdown of a key component of mTORC2, Rictor^41^ by shRNA, inhibited pT346-NDRG1 and Cox2 expression in fibroblasts treated with recombinant CXCL2/5 proteins or CM (0% FBS) from Panc02.shCtrl cells (Fig. 4j; Supplementary Fig. S10m). We further examined whether NLK and Yap1, upstream of CXCL2/5, in PDAC cells are critical for inducing Cox2 in hPSCs/fibroblasts. CM from Gem-treated Panc02.sh*NLK* cells and Capan1.shYap1 cells (0% FBS) significantly reduced Cox2 expression in hPSCs/fibroblasts compared to CM from corresponding control PDAC cells (Supplementary Fig. S10n, o). To investigate whether Gem treatment activates the Yap1-Cox2 cascade in vivo, we stained Yap1, CXCL5, and Cox2 in Vehicle- and Gem-treated KPC and KPC-*ζ*^*fl/fl*^ tumors. Gem treatment increased cancer cell nuclear Yap1, CXCL5, and fibroblast Cox2 in KPC tumors, while *14-3-3ζ* knockout diminished Gem-induced nuclear Yap1, CXCL5, and Cox2 expression (Fig. 4k, l). Together these results indicate that, in 14-3-3ζ+++ cancer cells, low-nutrient- or chemotherapy-induced stresses stabilize NLK protein to induce pS128-Yap1 that releases 14-3-3ζ-bound Yap1 for nuclear translocation and activation, leading to CXCL2/5 upregulation. This activates the CXCR2-mTORC2-Cox2-PGE2 axis in PSCs/fibroblasts, which conversely foster PDAC cell proliferation and survival under stress and Gem-treatment (Fig. 4m).

### Human PDAC Yap1 activation and stromal Cox2 expression

**Fig. 5:**
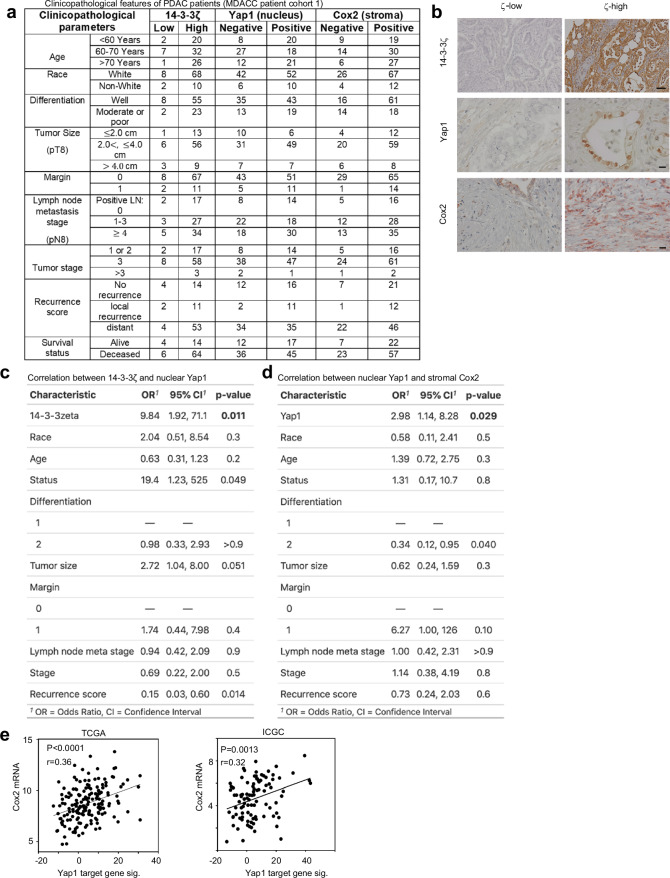
Analyses of the 14-3-3ζ-Yap1-Cox2 axis and associated clinicopathological characteristics in human PDACs. **a** Comparison of clinicopathological features of 113 patients with pancreatic cancer (MDACC patient cohort 1) expressing different levels of 14-3-3ζ (low vs high), nuclear Yap1 (negative vs positive), and stromal Cox2 (negative vs positive). **b** Representative IHC images of human PDAC tumors stained with the indicated proteins. scale bar: 50 µm (14-3-3ζ), 20 µm (Yap1 and Cox2). **c** Multivariate logistic regression analysis of the correlation between Yap1 (nuclear) and 14-3-3ζ by adjusting for the indicated clinicopathological characteristics. Odds ratio, 95% confidence intervals, and *P* values are presented. **d** Multivariate logistic regression analysis of the correlation between Cox2 (stroma) and Yap1 (nuclear) by adjusting for the indicated clinicopathological characteristics. Odds ratio, 95% confidence intervals, and *P* values are presented. **e** Correlation analysis between Yap1 target gene signatures and Cox2 gene expression using TCGA (Left) and ICGC (Right) PDAC patient datasets.

To examine the clinical relevance of our above findings, we stained 14-3-3ζ, Yap1, and Cox2 in patient PDAC tissues using IHC and analyzed clinicopathological relationships (Fig. 5a, b). Multivariate logistic regression analysis was performed by adjusting for multiple clinical and pathological parameters, including race, age, survival status, differentiation, tumor size, tumor margin, lymph node metastasis stage, tumor stage, and recurrence score (Fig. 5a–d). High expression of 14-3-3ζ was correlated with nuclear Yap1, and high nuclear Yap1 level was associated with stromal Cox2 expression after adjustment for other covariates (Fig. 5b–d, *P* = 0.011 for 14-3-3ζ with Yap1 as response variable, *P* = 0.029 for Yap1 with Cox2 as response variable). Consistently, Yap1-target gene signatures were associated with increased Cox2 expression in TCGA and ICGC datasets (Fig. 5e), reinforcing the clinical relevance and critical function of the symbiotic signaling between PDAC cells (Yap1) and fibroblasts (Cox2) in human PDACs.

### Co-targeting Yap1 and Cox2 with clinically applicable drugs renders PDACs vulnerable to Gem

**Fig. 6:**
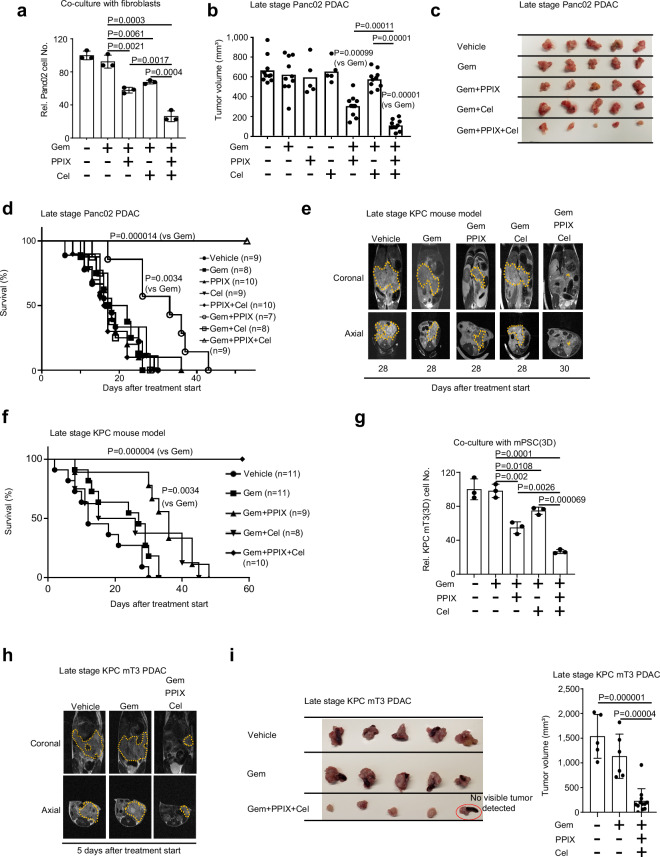
Co-targeting Yap1 in PDAC cells and Cox2 in fibroblasts by clinically applicable drugs boosts Gem response in mice with late-stage PDACs. **a** Relative cell number of Panc02 cells co-cultured with NIH3T3 cells in the presence of Gem (8.5 nM), Gem+PPIX (5 µM), Gem+ Cel (4 nM), or Gem+PPIX+Cel for 48 h. Panc02:NIH3T3 = 1:9. (mean ± SD, *t*-test, *n* = 3 biological repeats). **b** Tumor volumes of late-stage Panc02 tumors (day 18 after intrapancreatic injection) in mice 1 week after the indicated treatments (*n* = 10: Vehicle, Gem, Gem+PPIX, Gem+Cel, and Gem+PPIX+Cel; *n* = 5: PPIX, Cel, mean, Mann–Whitney test). **c** Images of treated Panc02 tumors quantified in **b**. **d** Kaplan–Meier survival analysis of mice bearing orthotopic Panc02 late-stage PDACs with indicated treatments (*n* = 9: Vehicle, *n* = 8: Gem, *n* = 10: PPIX, *n* = 9: Cel, *n* = 10: PPIX+Cel, *n* = 7: Gem+PPIX, *n* = 8: Gem+Cel, *n* = 9: Gem+PPIX+Cel. (Log-Rank test). **e** Representative MRI of KPC mice with the indicated treatments and times (i.e., 28 or 30 days after starting treatment). Yellow dotted circles indicate PDAC tumors. **f** Kaplan–Meier survival analysis of KPC mice bearing late-stage PDACs with the indicated treatments (*n* = 11: Vehicle, *n* = 11: Gem, *n* = 9: Gem+PPIX, *n* = 8: Gem+Cel, *n* = 10: Gem+PPIX+Cel). The treatment started at 55 days of age (Log-Rank test). **g** Relative cell number of KPC mT3 cells 3D-cultured in the lower chambers of a Transwell unit with 3D-cultured mPSCs in the upper chambers of a Transwell unit treated with vehicle, Gem (20 nM), Gem+PPIX (5 µM), Gem+Cel (4 nM), or Gem+PPIX+Cel for 72 h (mean ± SD, *t*-test, *n* = 3 biological repeats). **h** Representative MRI images of mice bearing KPC mT3 tumors with the indicated treatments (imaged 5 days after starting treatment). Yellow dotted circles indicate PDAC tumors. **i** Images (left) and quantifications (right) of tumor volume of late-stage (15 days after injection of KPC mT3 cells into the pancreas) PDACs after 7 days of the indicated treatments (*n* = 5: Vehicle, *n* = 6: Gem, and *n* = 12: Gem+PPIX+Cel, mean ± SD, *t*-test).

The clinical relevance data and the substantially enhanced Gem response in our proof-of-concept studies (Fig. 1h–k) inspired us to further assess the efficacy of co-targeting Yap1 and Cox2 in clinically relevant late-stage PDAC models using clinically used or tested drugs. Initially, we tested PPIX^23^, a clinically applicable drug targeting TEAD-Yap1 transcriptional activity, and Cox2 inhibitor Cel in a Panc02 PDAC model. Under co-culture with fibroblasts, Panc02 cell proliferations were modestly inhibited by treatments with Gem (< 8%), Gem+PPIX (42%), or Gem+Cel (32%) but strongly inhibited by Gem+PPIX+Cel (74%) treatment (Fig. 6a). In mice bearing Panc02 PDACs, Gem, PPIX, or Cel single treatments were ineffective starting from 18 days post-intrapancreatic injection, when mice had late-stage, palpable tumors and ascites (Fig. 6b–d; Supplementary Fig. S11a). Gem+PPIX treatment mildly delayed tumor growth and extended survival while Gem+Cel treatment was futile (Fig. 6b–d; Supplementary Fig. S11a), mirroring a failed phase II clinical trial of Gem+Cel in late-stage PDAC^42^. By contrast, co-targeting Yap1 with PPIX and Cox2 by Cel resulted in an exceptional Gem response, durable disease control (Fig. 6b, c), and an almost tripled median survival compared to that of Gem-treatment (57 days vs 19.5 days) (Fig. 6d; Supplementary Fig. S11a). Importantly, the co-targeting therapy was well tolerated by all the treated mice and did not induce discernable toxic effects based on blood urea nitrogen (BUN), aspartate transaminase (AST), and alanine transaminase (ALT) levels (Supplementary Fig. S11b). Together, data from co-targeting with clinically applicable drugs echo findings from genetically targeting Yap1 plus Cox2 inhibition by Cel (Fig. 1h–k), indicating the clinical potential of blocking the identified symbiotic signaling circuit between PDAC cells and fibroblasts.

To test the general efficacy of the co-targeting strategy on late-stage PDAC, we further assessed the therapeutic effects of various treatments in KPC (*P48-Cre*;*LSL-Kras*^*G12D*^;*Trp53*^*fl/fl*^) mice at 55 days of age, when they had developed advanced PDACs with a median survival of 64 days^43^. The KPC mice were randomized into five treatment groups and monitored for PDAC growth by magnetic resonance imaging (MRI). MRI detected large tumors in KPC mice treated with vehicle, Gem, Gem+PPIX, and Gem+Cel at 28 days after treatment but hardly detected any tumor in the Gem+PPIX+Cel-treated mice at 30 days with treatment (Fig. 6e). Strikingly, the median survival of Gem+PPIX+Cel-treated mice was tripled compared to Gem-treated mice (81.5 vs 27 days after treatment) (Fig. 6f; Supplementary Fig. S11c). Thus, co-targeting Yap1 and Cox2 with clinically applicable and well-tolerated agents produces unparalleled control of late-stage PDACs in KPC mice under Gem-treatment.

Approximately 50%–75% of human PDACs harbor *p53* mutations and 2% of PDACs have homozygous *p53* deletions^15^. *p53* mutation status in PDACs significantly impacts the stroma^44,45^, immune landscapes^46^, and response to chemotherapies^47^ and immunotherapies^46^. The mouse p53 R172H mutation corresponds to the most common p53 R175H mutation in human PDACs^15^; thus, we tested whether the co-targeting strategy also inhibits PDACs from KPC mT3 cells harboring the p53 R172H mutation. First, co-targeting Yap1 and Cox2 significantly inhibited KPC mT3 cells 3D co-cultured with mPSCs compared to other treatments (Fig. 6g). Next, C57BL/6 mice were given intrapancreatic injections of KPC mT3 cells and treated with Gem or Gem+PPIX+Cel from 8 days after injection, when the mice had developed late-stage, palpable tumors. Again, Gem alone was ineffective, but Gem+PPIX+Cel potently inhibited PDAC growth (Fig. 6h,i). Altogether, the symbiotic signaling circuit of 14-3-3ζ-Yap1-CXCL2/5-Cox2-PGE2 between PDAC cells and stromal fibroblasts is essential for PDAC adaptation to chemo-induced stress and blocking the circuit with clinically applicable drugs drastically enhanced chemotherapy response in all three clinically relevant late-stage PDAC models.

To examine whether the symbiotic circuit functions in vivo and blocking the circuit underlies the remarkable efficacy of the co-targeting strategy on late-stage PDACs, we collected PDAC samples from Gem, PPIX, and/or Cel-treated Panc02 tumors (Fig. 6b) and performed IHC analyses. Consistent with our in vitro findings, Gem-treatment, as a stressor, triggered the adaptive response characterized by increased nuclear Yap1 and high CXCL5 expression in cancer cells, upregulated stromal Cox2 and IGFBP2 (a fibroblast activation marker), and significantly more Ki-67-positive proliferating PDAC cells, all of which were markedly reduced by co-targeting Yap1 with PPIX and Cox2 with Cel (Supplementary Fig. S11d–i). These data confirmed the critical functions of the symbiotic signaling circuit of 14-3-3ζ-Yap1-CXCL2/5-Cox2 in the Gem-induced adaptive response of 14-3-3ζ+++ PDACs and the effective blockade of the circuit in late-stage PDACs by the co-targeting strategy in vivo.

### Co-targeting therapy reshapes the fibrotic TME of PDACs

**Fig. 7:**
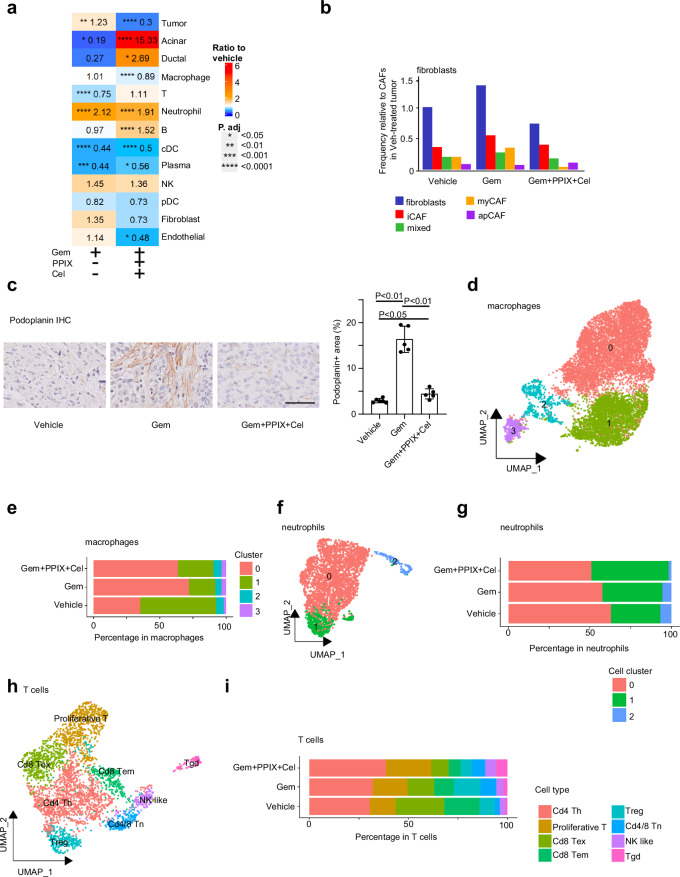
Co-targeting treatment reshapes the PDAC tumor microenvironment. **a** Normalized fold change of each cell type in Gem- or Gem+PPIX+Cel-treated KPC mT3 PDACs compared to vehicle-treated KPC mT3 tumors. Statistically significant changes based on Fisher exact test *P* values are indicated. **b** Frequencies of total fibroblasts and each fibroblast (sub)cluster in Vehicle-, Gem-, or Gem+PPIX+Cel-treated KPC mT3 tumor samples. **c** IHC staining of podoplanin in KPC mT3 PDACs with the indicated treatments (mean ± SD, *t*-test, *n* = 5–6 biological repeats, scale bar: 50 µm). **d** UMAP visualization of the 7282 macrophage cells from vehicle-treated, Gem-treated, and co-targeting therapy (Gem+PPIX+Cel)-treated KPC mT3 tumor sample. Color coding indicates the major (sub)clusters. **e** The fractions of each macrophage (sub)cluster in KPC mT3 tumors receiving the indicated treatments. **f** UMAP visualization of the 1942 neutrophils from vehicle-treated, Gem-treated, and Gem+PPIX+Cel-treated KPC mT3 tumor samples. Color coding indicates the major (sub)clusters. **g** The fractions of each neutrophil (sub)cluster in KPC mT3 tumor samples receiving the indicated treatments. **h** UMAP visualization of the 2695 T cells from vehicle-, Gem-, and Gem+PPIX+Cel-treated KPC mT3 tumor samples. Color is coded by major (sub)clusters. **i** The fractions of each T cell (sub)cluster in KPC mT3 tumor samples receiving the indicated treatments.

The striking responses to co-targeting therapy in late-stage PDACs illustrate the critical function of the fibrotic TME in PDAC Gem resistance. Also, various immune cell populations in the PDAC TME have been implicated in modulating therapeutic responses^48^. To systematically examine the global impact of co-targeting therapy on the PDAC stroma and immune microenvironment, tumor tissues were collected from vehicle-, Gem-, and Gem+PPIX+Cel-treated mice bearing KPC mT3-induced PDACs for scRNA-seq analyses (Fig. 7; Supplementary Figs. S12, S13). Uniform manifold approximation and projection (UMAP) showed diverse immune, stromal, and epithelial cell populations in late-stage KPC mT3 tumors, with tumor-associated macrophages (TAMs) as major immune cells in the TME (Supplementary Fig. S12a–c). Seurat analysis identified significant reductions in malignant KPC mT3 tumor cells by co-targeting therapy compared to vehicle and Gem treatments (Fig. 7a). Also, more acinar and ductal cells were found in the co-targeting therapy-treated KPC mT3 PDACs (Fig. 7a). Similar to a previous finding^49^, Gem-treatment of KPC mT3 PDACs increased fibroblasts, including inflammatory cancer-associated fibroblasts (iCAFs) and myofibroblastic CAFs (myCAFs), relative to the vehicle-treated group, whereas co-targeting treatment reversed the effects (Fig. 7b). This finding was further confirmed by IHC staining of podoplanin, a pan-CAF marker of PDAC^50^ (Fig. 7c). Gem-treatment significantly increased *Chil3*^*+*^ subset (cluster 0) and reduced *C1q*^*+*^ subset (cluster 1) in TAMs, but co-targeting therapy had no significant additional influence on TAM subsets (Fig. 7d, e; Supplementary Fig. S13a, b). Also, Gem-treatment had prominent effects on other myeloid cells, including increased ( ~2×) neutrophils and reduced ( ~50%) dendritic cells (DCs) compared to vehicle treatment (Fig. 7a). Co-targeting therapy did not further enhance neutrophil infiltration in KPC mT3 tumors than Gem-treatment, but co-targeting increased the IFN-γ-responsive and *Sell*^+^ neutrophil subset (cluster 1, Fig. 7f, g; Supplementary Fig. S13c–e), which was recently shown to be involved in anti-tumor activities and response to immunotherapies^51,52^. Additionally, co-targeting therapy moderately increased total B and T lymphocytes compared to Gem-treatment (Fig. 7a; B cells: 1.52 vs 0.97; T cells: 1.1 vs 0.75; the numbers indicate the ratio relative to the vehicle controls). Notably, Gem-treatment significantly reduced antigen-experienced CD8^+^ T cells, including exhausted T cells (Tex), effector memory T cells (Tem), and plasma cells (or effector B cells), indicating suppression of adaptive immune response by Gem (Fig. 7h, i; Supplementary Fig. S13f, g). Interestingly, co-targeting therapy reduced regulatory T (Treg) cells compared to vehicle treatment, whereas Gem-treatment increased Treg cells (Fig. 7i; Supplementary Fig. S13f). Co-targeting treatment also increased NK-like T cells and γδ T cells (Fig. 7i; Supplementary Fig. S13f). Together, the scRNA-seq data indicate that co-targeting Yap1 and Cox2 reshapes the late-stage PDAC TME, especially the fibrotic TME, which may contribute to enhanced Gem response.

### PDAC patients taking Statins and Cox2 inhibitors have significantly improved clinical outcome

**Fig. 8:**
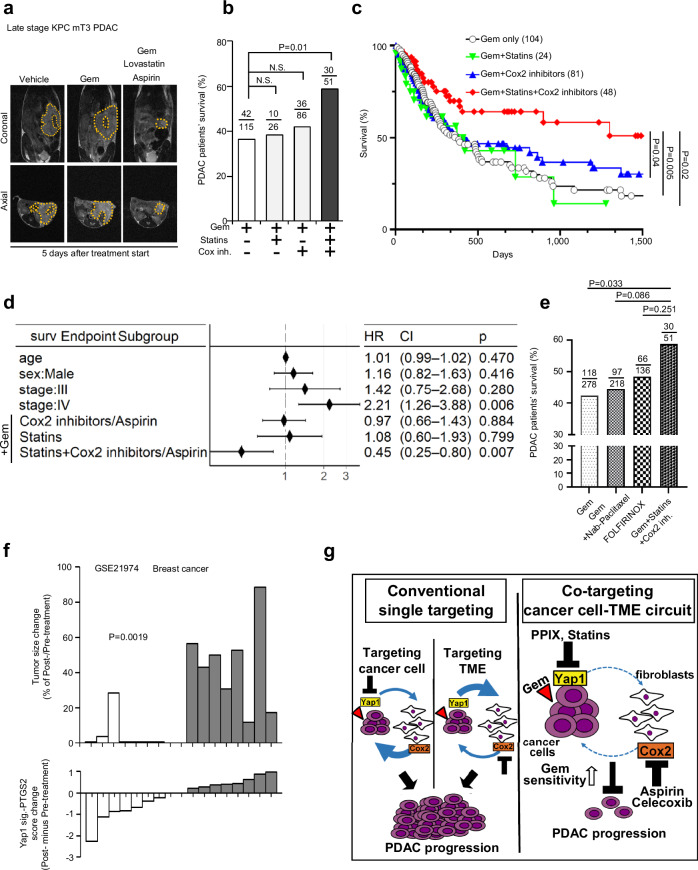
PDAC patients taking Yap1 inhibitor (Statins) and Cox2 inhibitors during Gem-treatment have significantly improved clinical outcomes. **a** Representative MRI images of mice bearing KPC mT3 tumors treated with the indicated drugs (5 days after treatment start date). Yellow dotted circles indicate PDAC tumors. **b** Survival rates of different PDAC patient groups (MDACC patient cohort 2) who took moderate Statin therapies, Aspirin/Cox2 inhibitors, or neither while receiving Gem treatment. The numbers refer to the surviving patients vs the total patients in each group. **c** Univariate survival analysis of the PDAC patient groups that received different treatments as indicated (log-rank test). **d** Multivariate survival analysis of indicated PDAC patient groups that received different treatments as indicated. **e** Survival rates of patients receiving Gem, Gem+Nab-Paclitaxel, FOLFIRINOX, or Gem+Statins+Aspirin/Cox2 inhibitors. The numbers refer to the surviving patients vs total patients in each group. **f** Analysis of tumor volume changes (%) after chemotherapy (Top) and changes of Yap1+PTGS2 signature score (Bottom) in corresponding breast cancer patients (accession number GSE21974). Each bar represents one patient. Only patients with significant changes in Yap1+PTGS2 signature scores ( < –0.2 or > 0.2) are presented here (*n* = 15, *t*-test *P* = 0.0019). **g** Compared to targeting only cancer cells or the TME, simultaneously co-targeting Yap1 in cancer cells and Cox2 in fibroblasts leads to an exceptional Gem response and durable disease control.

The staggering efficacy (Fig. 6) and global impact on the TME (Fig. 7) of the co-targeting strategy in late-stage PDAC mouse models implied its clinical potential for treating late-stage PDACs in patients. However, many PDAC experimental therapies showed impressive efficacy in mouse models but failed in clinical trials devastatingly, suggesting discrepancies between mouse models and patients in the clinic. Therefore, the most critical question is whether our co-targeting strategy is clinically effective in PDAC patients. We sought to examine whether PDAC patients treated with medications that block Yap1 signaling and Cox2 simultaneously during Gem-treatment may have better clinical outcomes compared to patients who were not treated with Yap1 and/or Cox2 blocking agents. Although no Yap1-specific inhibitor has been approved for treating cancer patients, common Statins can target the mevalonate pathway to block Yap1 nuclear localization and Yap1 target gene expression in PDAC cells in vitro and in vivo^53^. Indeed, Lovastatin, a common Statin completely abolished Gem-induced upregulation of Yap1 target genes (e.g., *CTGF* and *CYR61*) and CXCL2/5 expression in Panc02 PDAC cells (Supplementary Fig. S14a). Likewise, Aspirin, the most widely used non-steroidal anti-inflammatory drug (NSAID), is well-known to target Cox2 and significantly reduces the risk of Cox2-overexpressing colorectal cancers^54^. Lovastatin combined with Aspirin or Cel greatly improved the Gem response of PDAC cells co-cultured with PSCs/fibroblasts but had no effect without Gem treatment (Supplementary Fig. S14b, c). Importantly, Gem+Lovastatin+Aspirin combination treatment strongly inhibited KPC mT3-induced late-stage PDACs in vivo (Fig. 8a; Supplementary Fig. S14d). Therefore, we postulated that PDAC patients treated with Statins plus Cox2 inhibitors/Aspirin during Gem-treatment would have improved clinical outcomes compared to patients who did not receive Statins and Cox2 inhibitors/Aspirin. To test this, we retrospectively analyzed the electronic health record (EHR), via Epic Slicerdicer, of patients with advanced PDAC (stages II, III, and IV) treated at MDACC between 2016 and 2021. Among Gem-treated patients, those who simultaneously took Statins and Cox2 inhibitors (including Aspirin) had the best overall survival rate (59%), compared to the other three groups (Gem only: 37%; +Statins: 39%; +Cox2 inhibitors: 41%) (Fig. 8b). Furthermore, patients who took Statins and Cox2 inhibitors while receiving Gem-treatment showed significantly improved median survival (1507 days) (Fig. 8c), compared to other groups (Gem only: 383 days; +Statins: 420 days; +Cox2 inhibitors/Aspirin: 430 days). Multivariate survival analysis confirmed that the overall survival differences were independent of gender, age, and tumor stage of patients (Fig. 8d). Additionally, patients who took Statins and Cox2 inhibitors while receiving Gem showed a trend toward better survival outcomes compared to those who received combinatorial chemotherapy with albumin-bound paclitaxel (Nab-paclitaxel) plus Gem, which is the current standard therapy for PDAC patients, as well as better survival outcomes than those who received FOLFIRINOX, the most powerful chemotherapy regimen for treating advanced PDAC (Fig. 8e). Clearly, our analyses of patient EHR data echo our preclinical findings: co-targeting of Yap1 and Cox2 significantly boosts Gem response in advanced PDAC with exceptional therapeutic efficacy in multiple mouse models and significantly improved clinical outcome in PDAC patients.

### Adaptive upregulation of Yap1 and Cox2 in chemotherapy resistance beyond pancreatic cancer

Our findings on the critical functions of Yap1 and Cox2 signaling circuits in the adaptive stress response and the efficacy of co-targeting Yap1 and Cox2 in late-stage PDAC led us to further explore whether the Yap1-Cox2 signaling circuit is operative in other cancer types besides PDACs. Strikingly, 22 of 31 cancer types (70%) in TCGA datasets showed positive correlations (Pearson coefficient *r* > 0.3) between Yap1 signature (51-gene core signature) and *PTGS2* (Cox2) mRNA expression (Supplementary Fig. S15a). Upregulated Yap1 signature was also detected in patient-matched post-chemotherapy breast cancer tissues (Supplementary Fig. S15b, accession numbers GSE21974 and GSE4382), suggesting that Yap1 signaling activation is also an adaptive response to chemotherapies in breast cancers, similar to that in PDAC. Among breast cancer patients who received chemotherapy, none of eight patients with increased Yap1-Cox2 signature scores had a pathological complete response (pCR), while five of seven patients with lower Yap1-Cox2 signature scores had pCR (Fig. 8f), suggesting that increased Yap1-Cox2 signaling is also associated with adaptive resistance to chemotherapies in these patients.

## Discussion

In this study, we identified a symbiotic signaling loop between PDAC cells (14-3-3ζ-Yap1-CXCL2/5) and CAFs (CXCR2-mTORC2-Cox2-PGE2) that is critical for PDAC adaptive response to stress, including chemotherapy (Fig. 4m). The signaling circuit is initiated by stress-induced Yap1 activation in 14-3-3ζ+++ PDAC cells. It was well established that 14-3-3ζ binds with pS127-Yap1 to sequester Yap1 in the cytoplasm^55^. However, recent studies suggest that osmotic stress increases NLK phosphorylation^56^ to increase pS128-Yap1, which blocks 14-3-3 binding with Yap1, thus enhancing Yap1 nuclear accumulation^34^. Here, we report that different stressors (chemotherapy and nutrient deficiency) rapidly (< 5 min) inhibit NLK degradation in PDAC cells, increase pS128-Yap1, and trigger Yap1 nuclear accumulation, suggesting that NLK is a critical molecular switch for Yap1 activation via a non-canonical pathway under certain stresses. Interestingly, we found that stresses induced similar levels of NLK and Yap1-S128 phosphorylation in 14-3-3ζ+++ and 14-3-3ζ+ PDAC cells. Additionally, NLK overexpression failed to restore stress-induced Yap1 nuclear translocation in 14-3-3ζ+ PDAC cells. These data indicate that only NLK upregulation with increased pS128-Yap1 is insufficient to drive Yap1 nuclear translocation, 14-3-3ζ+++ is also required for stress-induced Yap1 nuclear accumulation. The required 14-3-3ζ+++ for stress-induced Yap1 nuclear accumulation is seemingly controversial to the well-known function of 14-3-3ζ as a “suppressor” of Yap1 nuclear translocation and activation via binding with pS127-Yap1 in the cytosol^33^. In fact, our data support the classic role of 14-3-3ζ in retaining Yap1 in the cytosol as we detected significant Yap1 and 14-3-3ζ binding in 14-3-3ζ+++ cells without stressors but their binding was reduced by stressors (Fig. 2b, c) which induced NLK upregulation and pS128-Yap1 (Fig. 2d), leading to increased Yap1 nuclear accumulation. Our data indicate that in NLK-mediated stress response of 14-3-3ζ+++ PDAC cells, 14-3-3ζ functions as a critical Yap1 adaptor that binds or releases Yap1 depending on the Yap1 phosphorylation status (pS127 vs pS128), thus makes Yap1 readily accessible in response to NLK-mediated Yap1-S128 phosphorylation to release 14-3-3ζ-bound Yap1 for nucleus translocation afterward. Conceivably, in 14-3-3ζ+sh*ζ* PDAC cells, Yap1 is not sequestered by 14-3-3ζ+ but might be sequestered in the cytosol by other Yap1 regulator(s) which have been reported to bind Yap1 independently of its phosphorylation status, i.e., AMOTL1/2^57^, SHP2^58^, IFT^59^. How 14-3-3ζ cooperates with upregulated NLK to enhance Yap1 nuclear translocation in 14-3-3ζ+++ cells warrants further investigation.

Importantly, Yap1 is emerging as a key signaling node involved in multiple adaptive cellular stress responses. Recent studies also found that Yap1 signaling orchestrates adaptive resistance to KRAS inhibitors, which further supports Yap1 as an attractive target for combinatorial therapies to treat refractory cancers^60,61^. Since chemotherapy is the most common treatment for PDAC patients^2^, targeting chemo-induced Yap1 adaptive stress response circuits has clear clinical potential as a treatment strategy.

Simultaneously co-targeting key nodes in PDAC cells (Yap1) and stromal inflammatory fibroblasts (Cox2) enhanced Gem therapeutic response without increasing adverse effects (Fig. 8g; Supplementary Fig. S11b), and this approach has an underlying mechanism fundamentally different from recent findings of improved chemo-response by dual targeting of Yap1 and Cox2 in colon and bladder cancers^26,27^. In colon or bladder cancer cells, overexpressing Yap1 induced Cox2 activation within cancer cells and triggered intrinsic resistance that was blocked by targeting Yap1 and/or Cox2 solely in cancer cells^26^. Differently, in PDACs, stress induces the Yap1 and Cox2 signaling circuits via cancer cells interacting with stromal fibroblasts, which requires co-targeting Yap1 in cancer cells and Cox2 in fibroblasts to intervene. Also, the stress-induced Yap1-Cox2 signaling circuit is an adaptive response, as co-targeting Yap1-Cox2 without Gem-treatment did not significantly impact late-stage PDACs (Fig. 6d). Further, inhibiting Yap1 or Cox2 singly does not make PDAC vulnerable to Gem-treatment, indicating that targeting cancer cells only or tumor stroma alone is insufficient to blackout the chemo-induced symbiotic signaling (Fig. 8g, left). This partly explains the failures of previous clinical trials that solely targeted cancer cells or stroma. The Yap1-Cox2 signaling also seems to be generally associated with adaptive chemo-resistance in other cancers (Fig. 8f; Supplementary Fig. S15a, b). Thus, co-targeting the symbiotic signaling of cancer cells and TME may confer robust anti-tumor efficacy for treating late-stage PDACs and other aggressive cancers. In supporting that, PDAC patients who took Statins and Aspirin/Cox2 inhibitors while receiving Gem-treatment had significantly improved overall survival compared to patients who received other treatments (Fig. 8b–e). Once these findings are validated in additional patient cohorts and further tested in prospective clinical trials, the co-targeting strategy can be rapidly employed to efficaciously treat patients with advanced PDACs, an unmet medical need. Since the co-targeting strategy is well-tolerated in pre-clinical models and agents for blocking Yap1 and Cox2 signaling (PPIX, Lovastatin, Cel, and Aspirin) are clinically applicable drugs with low toxicity, co-targeting may also provide alternative therapies for PDAC patients who are vulnerable to immunotherapy-induced toxicities and autoimmune responses or do not respond to current standard PDAC therapies, such as Nab-paclitaxel+Gem and FOLFIRINOX^62^ (Fig. 8e).

To fully understand how co-targeting disrupts the symbiotic circuit between PDAC cells and stroma to enhance therapeutic response, it is critical to dissect the dynamic interactions between tumor cells and their TME in response to treatments. Our scRNA-seq analyses of late-stage KPC mT3 tumors revealed the global impact of co-targeting therapy on PDAC stroma and the immune microenvironment, especially decreasing the number of fibroblasts and increasing the *Sell*^+^ neutrophil subset. Whether and how these alterations contribute to the remarkable efficacy of co-targeting therapy merits future exploration. Our scRNA-seq data also indicate that co-targeting treatment reduced iCAFs and myCAFs compared to Gem-treatment (Fig. 7b), which likely resulted from inhibiting Yap1, as pancreas-specific Yap1 knockout in KPC mice significantly reduced α-SMA^+^ (a myCAFs marker) stromal fibroblasts in PDACs^63^. Further understanding of mechanisms of disrupting cancer cells and TME by co-targeting strategy will foster its clinical potential.

## Materials and Methods

### Antibodies and reagents

PPIX was purchased from Sigma-Aldrich (Missouri, USA) and Santa Cruz Biotechnology (Texas, USA). Cel was purchased from LC laboratories (Massachusetts, USA). PGE2 was purchased from Sigma-Aldrich. MG132 was purchased from Selleck Chemicals (Texas, USA). Acetylsalicylic acid (aspirin) and Lovastatin were purchased from ACROS Organics (Massachusetts, USA). Gemcitabine hydrochloride was purchased from Santa Cruz Biotechnology and LC Laboratories. Cell culture inserts (12 wells, 1 µm pore size) were purchased from Greiner Bio-One (Kremsmünster, Austria). Recombinant mice CXCL2 and CXCL5 were purchased from R&D systems (Minnesota, USA). Antibodies to Yap1 (4912, 14074), P-Yap1 (Ser127, 4911), YY1 (2185), Cox2 (12282), P-NDRG1 (Thr346, 3217), Rictor (2140), NLK (94350), P-Akt (Ser473, 4060), Akt (9272), E-Cadherin (3195), Tyro3 (5585), and SF2 (14902) were purchased from Cell Signaling Technology (Massachusetts, USA). Antibodies to mouse CXCL5 (ab18134), mouse CXCL2 (ab25130), mouse podoplanin (ab256559), human CXCL2 (ab91511), human CXCL5 (ab126763), Ki-67 (ab16667), Cox2 (ab15191), and IGFBP2 (ab188200) were from Abcam (Cambridge, UK). Antibody to mouse CXCL5 (bs-2549R) was from Bioss antibodies (Massachusetts, USA). Antibodies to GAPDH (sc-32233), Yap1 (sc-101199), NDRG1 (sc-30040), IL-8RB (CXCR2, sc-683), 14-3-3ζ (sc-1019), and normal mouse IgG (sc-2025) were purchased from Santa Cruz Biotechnology. The tubulin antibody (T5168) was purchased from Sigma-Aldrich. The blocking antibodies to mouse CXCL2 (MAB452), mouse CXCL5 (MAB433), human CXCL5 (MAB254), and control antibody (MAB0061) were purchased from R&D Systems and human CXCL2 (311001) was purchased from Biolegend (California, USA). HA antibody (11667149001) was purchased from Roche (Indiana, USA). P-Ser128-Yap1 antibody was obtained from Dr. Eek-hoon Jho (University of Seoul).

### PGE2 enzyme-linked immunosorbent assay

PGE2 in CM was detected using the Prostaglandin E2 Parameter Assay Kit (R&D systems) following the manufacturer’s instructions.

### Cell lines, plasmids, shRNA, and siRNA

Mouse PDAC, (Panc02), human PDAC (Capan-1, MDA-Panc-28, PANC-1), and immortalized human pancreatic duct epithelial (HPDE-E6E7) cell lines were obtained from Dr. Paul J. Chiao (MDACC), KPC mT3 cells from Dr. Anirban Maitra (MDACC), hPSCs from Dr. Rosa Hwang (MDACC), iKras.sgCtrl/sg*Yap1* cells and PATC53 cells from Dr. Haoqiang Ying (MDACC), NIH3T3 cells from American Type Culture Collection (ATCC), and human pericytes (hPC-PL) from Promo Cell (Heidelberg, Germany). Primary mPSCs were isolated from the normal pancreas of 7–8-week-old C57BL/6 mice by the previously described method^64^. KPC cells were established from PDAC tumor tissue from KPC mice as previously described^65^, with minor modifications. For standard cell culture conditions, the cell lines were cultured in DMEM/Ham’s F-12 medium supplemented with 10% FBS and 1% penicillin/streptomycin. PDAC cells and PSCs in 3D-culture were prepared as previously described^20,66^ using growth factor-reduced matrigel (Corning, New York, USA) and Cultrex Reduced Growth Factor Basement Membrane Extract (R&D systems). shRNA clones were obtained from Sigma-Aldrich or Open Biosystems (Alabama, USA). Mouse *14-3-3ζ* shRNA, human *14-3-3ζ* shRNA, mouse *Yap1* shRNA, human *Yap1* shRNA, and human HA-14-3-3ζ overexpressing vectors were described previously^67,68^. Mouse *CXCL2* shRNA (clone ID: V3LMM_498125) and mouse *CXCL5* shRNA (clone ID: V2LMM_27698) were purchased from Open Biosystems. Mouse *CXCR2* shRNAs (clone IDs: TRCN0000421540 and TRCN0000432429), mouse *Rictor* shRNAs (clone IDs: TRCN0000123396 and TRCN0000123397), and mouse *NLK* shRNAs (clone IDs: TRCN0000226071 and TRCN0000218649) were purchased from Sigma-Aldrich. Doxy-inducible mouse *Yap1* shRNA and control shRNA were obtained from Dr. Guocan Wang (MDACC). Lentivirus particles for shRNAs and HA-14-3-3ζ overexpressing vectors were generated and transduced into target cell lines, and cell lines were selected with puromycin as previously described^67^. Human NLK (HG11573-UT) overexpressing vector was purchased from Sino Biological (Texas, USA). Yap WT and mutant vectors were obtained from Dr. Eek-hoon Jho (University of Seoul). Mouse *Yap1* (EMU088231) and *Cox2* (EMU039641) siRNAs were obtained from Sigma-Aldrich. For 14-3-3ζ rescue experiments, *14-3-3ζ* shRNA resistant transgene was designed and synthesized by Epoch Life Science (Texas, USA) and cloned into lentiviral plasmid pLV-EF1a-MCS-IRES-puro-GFP. PDAC sh*ζ* cells were transduced with the plasmid and GFP^+^ cells were sorted by flow cytometry for further analysis.

### In vivo experiments

All animal experiments were performed according to the approved animal protocols by the Institutional Animal Care and Use Committee of MDACC. KPC (*P48-Cre;LSL-Kras*^*G12D*^*;Trp53*^*fl/fl*^, FVB/C57BL/6 hybrid background) mice were obtained from Dr. Ronald A. DePinho (MDACC). To create *14-3-3ζ* conditional knockout mice, the ES cells with targeted Ywhaz (*14-3-3ζ*) allele were purchased from the International Knockout Mouse Consortium-European Conditional Mouse Mutagenesis (Eucomm). Three positive clones were selected and injected into the blastocyst. To generate floxed mice, chimera mice were then crossed with B6.Cg-Tg(ACTFLPe)9205Dym/J (Flp) mice that were purchased from Jackson Laboratory (Maine, USA). To generate the *14-3-3ζ* pancreas-specific knockout KPC mouse model (*P48-Cre;LSL-Kras*^*G12D*^*;Trp53*^*fl/fl*^*;14-3-3ζ*^*fl/fl*^), *14-3-3ζ*-floxed mice were crossed with KPC mice. In homozygous mice, exon 4 of Ywhaz (*14-3-3ζ*) is knocked out and 14-3-3ζ expression in the pancreas is ablated. Primers used in genotyping for *14-3-3ζ*-floxed mice are as follows:

forward, 5’-AGCACTGAGGTGCTGGCTAT-3’; reverse, 5’-CCAACAATACTGGGCATGTG-3’.

*Cox2* knockout mice were purchased from Taconic (New York, USA). C57BL/6 mice were purchased from MDACC Experimental Radiation Oncology Department. Syngeneic orthotopic PDAC tumors were generated by PDAC cell injection: 5 million cells (Panc02) or 0.5 million cells (KPC mT3) in 50 µL PBS were injected into the tail of the pancreas. For tumor volume analysis, the mice bearing tumors were euthanized, and tumors were excised on the indicated day. Tumor volume was measured and calculated using the following formula (length × width × height × 0.523).

For treatment experiments, mice were treated as follows: Gem (333 mg/kg) by intraperitoneal injection once a week, PPIX (8.26 mg/kg) by oral gavage administration three times a day, Cel (82 mg/kg) by oral gavage administration twice a day, Lovastatin (8.2 mg/kg) by oral gavage administration once a day, Acetylsalicylic acid (Aspirin, 41 mg/kg) by oral gavage administration once a day. The drug dosage used in mouse experiments was calculated based on the human clinical dosage^69^. Treatment started in 55-day-old KPC mice, 18 days after Panc02 cell injection (late-stage), and in C57BL/6 mice bearing orthotopically injected KPC mT3 cells 8 days after injection. After 7 days of treatment, mice were euthanized and tumors were dissected. KPC mT3 tumor samples were used for scRNA-seq. For survival analysis, mouse lifespan was monitored during treatment. In vivo MRI (4.7 T small animal MRI system) was performed at the small animal imaging facility at MDACC. For inducible sh*Yap1* experiments in vivo, orthotopic PDAC tumors were generated by injection of Panc02.doxy-inducible sh*Yap1* and shCtrl cells into the tail of the pancreas. Mice were treated with doxy food and doxy water 16 days after Panc02 cell injection for 9 days (water) and 2 days (food). Tumor-bearing mice were euthanized at 25 days after injection, and tumors were excised. Blood urea nitrogen, aspartate transaminase, and alanine transaminase analyses were performed by MDACC research animal support facility.

### scRNA-seq

Mice were euthanized 15 days after injection of KPC mT3 cells into the pancreas, and PDAC tumors were collected. Five representative tumors with similar tumor sizes from each group were selected for sample preparation. From each tumor, 0.1–0.2 g of tumor tissue was collected and combined into one mixed sample. Based on the test, 0.15 g of tissue yielded about 1.5 million cells. Tumor tissues were minced into small pieces and then digested with i) collagenase D, 2.5 mg/mL (Sigma-Aldrich); ii) Dispase II, 2 mg/mL (Sigma-Aldrich); iii) DNase I, 0.2 mg/mL (Stem Cell Technology, Massachusetts, USA). Tissues were digested at 37 °C for 45 min. The digested tissues were passed through a 40 µm cell strainer and washed with 5 mL fresh medium; 5 mL red blood cell lysis buffer was added to each sample. Red blood cells were lysed for 5 min followed by neutralization with 5 mL fresh medium, centrifugation, cell counting, and making cell suspension at 1 million cells/100 µL in sterile PBS plus 0.5% BSA and 2 mM EDTA. Cells were stained with Viability Dye (eBioscience Fixable Viability Dye eFluor 450, 1 µL for 1 mL PBS) for 30 min on ice, washed with PBS plus 0.5% BSA, and then proceeded for cell sorting. 100 K viable cells were collected for each sample and suspended in 100 µL PBS with 0.04% BSA at 1000 cells/µL density and immediately proceeded for scRNA-seq. The scRNA-seq raw data were demultiplexed and aligned to the mm10 reference genome using Cell Ranger (v7.0.0). The filtered UMI count matrices from Cell Ranger output were analyzed using Seurat (v4.3.0)^70^. For quality control, cells with less than 200 genes or more than 20% mitochondrial gene counts were filtered. The filtered data were then normalized by library size and log_2_-transformed. Two thousand highly variable genes were selected and scaled for dimension reduction and clustering. Principal component analysis was performed on the highly variable genes, and the first 20 principal components were used for UMAP visualization. The same set of principal components was also used to construct nearest-neighbor graphs for clustering using the Louvain algorithm. Differential expression analysis was used to identify the marker genes for each cluster. To annotate individual clusters, we manually matched the top markers with canonical cell type markers or markers from reference scRNA-seq datasets listed on the Azimuth website^70^. Tumor cells were further confirmed by high copy number variation using results from InferCNV (v1.10.1, https://github.com/broadinstitute/inferCNV).

To identify cell subpopulations within major cell types, we performed separate analyses for macrophages, T cells, neutrophils, tumor cells, and fibroblasts. For each cell type, we first removed potential doublets that highly expressed markers of other cell types. Next, we selected 2000 highly variable genes specific to each cell type. These genes were used for the dimension reduction and clustering using procedures similar to those applied to the entire dataset. We performed differential expression analysis among clusters to obtain marker genes. The marker genes of some subpopulations were used for enrichment analysis to characterize functions using clusterProfiler (v4.2.2)^71^. In some cases, we referred to previous studies to annotate some cell subpopulations like T cells and tumor cells. For some subpopulations with reported signatures, the signature score was calculated as the average expression of signature genes.

To assess the proportional differences of cell types across different groups, we employed a Fisher exact test-based method. For each cell type within an experimental group (Gem or Gem+PPIX+Cel), a 2 × 2 contingency table was constructed, consisting of the number of that cell type in the experimental group and control group (Vehicle), as well as the total number of cells in each group. Fisher’s exact test was then performed to calculate the *P* value from the contingency table, and the Benjamini-Hochberg procedure was applied to adjust the *P* values obtained from all tests. Proportional analysis for cell subpopulations followed a similar approach, with cell numbers for each cell type replaced by cell numbers for each subpopulation, and the total cell number replaced by the cell number for that particular cell type.

### mRNA sequencing (mRNA-seq)

Total RNA was isolated from KPC mT3 (3D) cells treated with Vehicle vs Gem (20 nM) for 72 h using Trizol reagent (Invitrogen, California, USA) and was reverse transcribed using the iScript cDNA Synthesis Kit (Bio-Rad, California, USA) following the manufacturer instructions. mRNA-seq was performed at the UT Health Cancer Genomics Center. The RNA-seq raw FASTQ files were mapped to the mouse transcriptome (mm39) using Salmon (v0.14.1) and STAR2 (v2.6.0b) to produce raw gene read counts and normalized TPM (transcript per million) values. Differential gene expression analyses were performed using R packages DESeq2 and limma. Pathway analyses were performed using the R fgsea package and online Metascape resource (https://metascape.org/gp/index.html). We used a YAP signature from GSEA database MsigDB (https://www.gsea-msigdb.org/gsea/msigdb, CORDENONSI_YAP_CONSERVED_SIGNATURE). Gene signature scores were calculated as the sum of all MAD-modified *Z* scores from all signature genes.

### Cell growth and apoptosis assay

For cell growth assays, PDAC cells were seeded onto 6-well plates. The cells were trypsinized and counted using a light microscope. The apoptosis assay was performed using the Annexin-V-FLUOS Staining Kit (Roche Life Science, Penzberg, Germany) and the BD Pharmingen FITC Annexin V Apoptosis Detection Kit I (BD biosciences, New Jersey, USA) following the manufacturer instructions. For one-way interaction cell counting or apoptosis detection experiments, PDAC cells or fibroblasts were incubated in either 0% FBS DMEM/Ham’s F-12 medium or Gem in 10% FBS DMEM/Ham’s F-12 medium for 24–48 h. The CM was collected after the 24–48 h incubation period. PDAC cells or fibroblasts plated on 6-well plates (for cell growth assay) and 96-well plates (for apoptosis assay) were incubated with CM for 24–48 h, followed by cell number or apoptosis analysis. For two-way interaction cell growth or apoptosis detection experiments, PDAC cells were incubated under stress (0%–0.1% FBS or Gem) for 48–72 h. CM from PDAC cells was collected. Fibroblasts were incubated with CM from PDAC cells for 24–72 h. After incubation, CM from fibroblasts was collected. PDAC cells were cultured in CM from fibroblasts for 24–72 h, then the number of PDAC cells or apoptotic cells was counted. For co-culture experiments, GFP-expressing PDAC cells and mCherry-expressing fibroblasts (1:1 or 1:9) were seeded on 6-well plates. After exposure with stress (0%–0.5% FBS or Gem) for the indicated time, the cells were trypsinized and PDAC expressing GFP expressing PDAC cells were counted using a fluorescence microscope.

### Fractionation of cytoplasmic and nuclear proteins

PDAC cells were scraped off plates with PBS and collected into 1.5 mL tubes. After centrifuging at 3000 rpm for 5 min, PDAC cell pallets were re-suspended and incubated on ice with a cytoplasmic extraction buffer (10 mM HEPES pH 8, 1.5 mM MgCl_2_, 10 mM KCl, 200 mM sucrose, 2% NP-40, protease inhibitor cocktail) for 5 min. After centrifuging at 3000 rpm for 5 min, the supernatant (cytoplasmic extract) was collected. After PDAC cell pellets were washed twice with wash buffer (10 mM HEPES pH 8, 1.5 mM MgCl_2_, 10 mM KCl), the pellets were re-suspended and incubated on ice with a nuclear extraction buffer (10 mM HEPES pH 8, 1.5 mM MgCl_2_, 400 mM NaCl, 0.1 mM EDTA, 25% Glycerol, protease inhibitor cocktail) for 10 min. After centrifuging at 14,000 rpm for 5 min, nuclear extracts (and cytoplasmic extracts) were collected for WB analysis.

### WB

WB analyses were performed as previously described^67^. Protein samples were separated by SDS-PAGE and transferred onto a nitrocellulose membrane. The membrane was blocked with 5% milk TBST for 1 h at room temperature. The membranes were probed with various primary antibodies at 4 °C overnight, incubated with secondary antibodies for 1 h at room temperature, and developed with chemiluminescence reagents (ThermoFisher Scientific, Massachusetts, USA).

### IP

Cell lysates were prepared as previously described^67^. Cell lysates were diluted with IP buffer, and then, were incubated with Anti-HA magnetic beads (ThermoFisher Scientific) overnight at 4 °C. The beads were then pelleted and washed with cold IP buffer three times. The washed immunoprecipitated complex was mixed with 2× loading buffer (100 mM Tris-HCL (pH 6.8), 200 mM DTT, 4% SDS, 0.2% Bromophenol blue, 20% glycerol) at a 1:1 ratio and denatured by boiling for 5 min, followed by WB analysis with specific antibodies as described above.

### Quantitative real-time PCR

Total RNA was isolated from PDAC cells using Trizol reagent (Invitrogen) and was reverse transcribed using the iScript cDNA Synthesis Kit (Bio-Rad) following the manufacturer’s instructions. Real-time PCR was performed using template cDNA and iQ SYBR Green Supermix (Bio-Rad) by the StepOnePlus real-time PCR system (Applied Biosystems, Massachusetts, USA). The threshold cycles for target genes were normalized to the threshold cycles of GAPDH to analyze relative gene expression levels. Primers used in qRT-PCR experiments are as follows:

mouse *Cox2* forward, 5′-TGAGCAACTATTCCAAACCAGC-3′.

mouse *Cox2* reverse, 5′-GCACGTAGTCTTCGATCACTATC-3′.

mouse *CXCL2* forward, 5′-CCAACCACCAGGCTACAGG-3′.

mouse *CXCL2* reverse, 5′-GCGTCACACTCAAGCTCTG-3′.

mouse *CXCL5* forward, 5′-GTTCCATCTCGCCATTCATGC-3′.

mouse *CXCL5* reverse, 5′-GCGGCTATGACTGAGGAAGG-3′.

mouse *GAPDH* forward, 5′-AGGTCGGTGTGAACGGATTTG-3′.

mouse *GAPDH* reverse, 5′-TGTAGACCATGTAGTTGAGGTCA-3′.

mouse *NLK* forward, 5′-TGGGCAACAACAGCCATATTT-3′.

mouse *NLK* reverse, 5′-GTGCGCCTTAACTGTAGCAG-3′.

mouse *CTGF* forward, 5′-GGGCCTCTTCTGCGATTTC-3′.

mouse *CTGF* reverse, 5′-ATCCAGGCAAGTGCATTGGTA-3′.

mouse *CYR61* forward, 5′-CTGCGCTAAACAACTCAACGA-3′.

mouse *CYR61* reverse, 5′-GCAGATCCCTTTCAGAGCGG-3′.

human *CXCL2* forward, 5′-CGCCCAAACCGAAGTCATAG-3′.

human *CXCL2* reverse, 5′-GCCATTTTTCAGCATCTTTTCG-3′.

human *CXCL5* forward, 5′-AGCTGCGTTGCGTTTGTTTAC-3′.

human *CXCL5* reverse, 5′-TGGCGAACACTTGCAGATTAC-3′.

human *GAPDH* forward, 5′-ACAACTTTGGTATCGTGGAAGG-3′.

human *GAPDH* reverse, 5′-GCCATCACGCCACAGTTTC-3′.

### ChIP-qPCR assay

ChIP-qPCR assay was performed by using Zymo-Spin ChIP kit (Zymo Research, California, USA) following the manufacturer’s instructions. Briefly, 3D-cultured PANC-1 cells were treated with Gem (20 nM) for 24 h. Then, the cells were harvested and cross-linked with 37% formaldehyde (final, 1%), treated with glycine (final, 0.125 M), washed, resuspended in chilled chromatin shearing buffer, and sonicated. Lysates containing soluble chromatin were incubated with antibodies against Yap1 (Santa Cruz Biotechnology, sc-101199) or IgG (Santa Cruz Biotechnology, sc-2027) overnight at 4 °C and then incubated for an additional 1 h at 4 °C with added ZymoMag Protein A beads while rotating. The magnetic bead-bound complexes were then clustered with a magnetic stand and washed three times. Elution and reversal of the cross-linking of CHIP DNA was performed by incubating the magnetic beads with chromatin elution buffer and 5 M NaCl at 75 °C for 5 min, and then the eluate was incubated at 65 °C for 30 min and 90 min at 65 °C with proteinase K. ChIP DNA was purified using Zymo-Spin IC Column, and 1 μL of DNA was analyzed via qPCR using specific primers for the CXCL2 and CXCL5 promoters, which were identified by the NCBI database (https://www.ncbi.nlm.nih.gov/). Potential binding sites of Yap1 on *CXCL2* (NC_000004.12:c74097039-74095040) and *CXCL5* (NC_000004.12:c73995641-73993642) promoters were predicted by JASPAR (https://jaspar.genereg.net/). The ChIP assay primers used were as follows:

human *CXCL2* forward, 5′-CTGCATTAGTTTGCATCCTTCCT-3′;

human *CXCL2* reverse, 5′-CCTGGGGCAGCACTGAATAC-3′;

human *CXCL5* forward, 5′-AGTTATCCCTTCACAGCACCAA-3′;

human *CXCL5* reverse, 5′-TCAGGAATGGGATGAGACAGA-3′.

All fold-enrichment values were normalized to IgG.

### Cytokine/chemokine array

Panc02.shCtrl cells and Panc02.sh*ζ* cells were cultured in 0% FBS DMEM/Ham’s F-12 medium for 72 h, and the CM was collected and used in a cytokine/chemokine array analysis. The cytokine/chemokine array analysis was performed using Milliplex MAP Mouse Cytokine/Chemokine Magnetic Bead Panel following the manufacturer’s instructions (Millipore, Massachusetts, USA).

### IHC and IF staining

IHC and IF analyses were performed as previously described^67,68^. IHC slides were evaluated by three pathologists independently. For IF, confocal microscopy was done using Zeiss LSM710 (Zeiss, Oberkochen, Germany).

### Reverse phase protein array

RPPA was performed using the MDACC Functional Proteomics Core facility as previously described^67^.

### Human Subjects

All procedures in studies involving human subjects were performed under the ethical standards of the institutional research committee and the Helsinki Declaration or comparable ethical standards following the guidelines approved by the Institutional Review Board (IRB) at MDACC. In this study, we performed retrospective reviews of the institutional medical databases fed from the electronic health record. To protect patient confidentiality, deidentified patient data were included, and no Protected Health Information was collected. No treatments were given, and no subjects were contacted. It did not involve more than minimal risk to the subjects. The informed consent from the patients was therefore waived, and the request for Waiver of Informed Consent has been approved by the IRB committee. All patient-related data and unique identifiers were removed so that human information was anonymized before any further processing.

### EHR analysis

Our clinical data mining study was conducted on PDAC patients encountered from 2016 through 2021 at MDACC. The study population consisted of cancer patients with the International Classification of Diseases (ICD) code (10th version). Anonymized aggregate-level data were collected using the SlicerDicer function within MDACC’s Epic EHR. Institutional Review Board approval was therefore waived. Using the Epic SlicerDicer, we identified 9597 patients with a visit diagnosis, billing diagnosis, or active problem list with malignant neoplasm of the pancreas (ICD-10 Code: C25*). Among the 9597 patients, 1272 patients who were diagnosed with malignant neoplasm of the endocrine pancreas (ICD-10 code: C25.4) were removed and the remaining 8325 patients were mainly PDAC patients. We further identified 1818 patients who had the tumor stage information and were diagnosed with advanced stages (II, III, or IV) PDAC. Among these patients, 621 patients were treated with Gem based on the EHR. Many patients with advanced-stage PDAC were treated with multiple rounds of different combinatory therapies. To avoid the potential impact of other powerful therapies that could interfere with the analysis, we excluded the patients treated with FOLFIRINOX from the initial analysis. There were 279 patients with advanced-stage PDAC treated mainly with Gem. Patient information, including gender, age, tumor stage, Gem-treatment starting date, death date, and vital status (up to Dec. 31, 2021) was pulled out via Epic Slicerdicer. To analyze the impacts of Statins and Cox2 inhibitors on patient outcomes, patients were divided into four subgroups based on the status of Statins and Cox2 inhibitor uptake. Statin therapies can be further broken into three categories: low intensity, moderate intensity, and high intensity. Generally, Statin intensity refers to how powerful a Statin is to lower the low-density lipoprotein (LDL) cholesterol. High-intensity Statins are given to people most at risk for heart-related complications due to high cholesterol. Cancer patients receiving high-intensity Statins may have preexisting health conditions that may affect clinical outcomes during chemotherapy. To avoid a potentially biased selection of patients, patients receiving moderate-intensity Statin therapies were chosen for further analysis. The survival rate of each subgroup was calculated based on the number of surviving patients vs the total number of patients in the subgroup. Because most patients’ direct responses to therapy were not available in Epic SlicerDicer, the overall survival rate was used as a surrogate indicator of therapeutic response. Patients whose HER did not include the date of starting Gem-treatment were excluded from the survival analysis. The survival time was calculated as the days between the treatment starting date and the death date. The univariate survival analysis and multivariate survival analysis were performed using SPSS Statistics.

### Patient samples

PDAC patient specimens were collected at MDACC and processed in compliance with protocol (Lab05-0854) approved by the MDACC Institutional Review Board. Informed consent was obtained from all patients. For the Yap1 analysis, the patient samples with Yap1 low/negative PDAC tumors (less than score 100) were excluded.

### Bioinformatics and statistical analysis

Our list of the 668 unfavorable genes in pancreatic cancer was generated based on methods described at https://www.proteinatlas.org/humanpathology/pancreatic+cancer^10^. Briefly, transcriptomics data from TCGA pancreatic cancer patients with an indicated clinical outcome were retrieved for analysis (https://gdc-portal.nci.nih.gov/), and 1523 genes were suggested as prognostic based on transcriptomics data from 176 patients (668 genes associated with unfavorable prognosis and 855 genes associated with favorable prognosis). The prognostic genes were further validated by antibody-based IHC or IF staining.

RNA-seq and clinical metadata corresponding to TCGA human pancreatic cancers were retrieved from the Genomic Data Commons (GDC) Data Portal (https://gdc-portal.nci.nih.gov/). Based on FPKM (fragments per kilobase of exon per million reads) value of Ywhaz (*14-3-3ζ*) and PTGS2 (*Cox2*), patients were classified into two expression groups (14-3-3ζ high vs 14-3-3ζ low, Cox2 high vs Cox2 low), and the correlation between expression level and patient survival was examined.

TCGA RNA-seq data were downloaded from the Broad Institute Firehose website (https://gdac.broadinstitute.org/). ICGC RNA-seq data were downloaded from the ICGC website (https://dcc.icgc.org/projects/PACA-AU). Patient samples from TCGA and ICGC were separated into 14-3-3ζ expression low, medium, and high groups based on Kmeans 3 separation of log_2_-normalized YWHAZ expression values. GSEA analysis was performed using software downloaded from https://www.gsea-msigdb.org/gsea/index.jsp (MSigDB version 7.4) using expression data from 14-3-3ζ high and low samples. GSEA enrichment plots were drawn using the R fgsea package. For *Yap1* signature in PDAC, we used a 29-gene signature derived from a reported *Yap1* signature of 57 genes found in breast cancer^19^. These 29 genes show elevated expression in the squamous subtype of ICGC PDAC tumors, which have higher *Yap1* activation^72^. Therefore, we consider this subset of 29 genes to be more relevant to PDAC tumors. Gene signature scores were calculated by summation of MAD-based Z scores of all component genes.

For pan-cancer analysis of the *Yap1* signature, a reported *Yap1* signature of 57 genes found in breast cancer^19^ was adopted. RNA-seq data were downloaded from the TCGA website, and *Yap1* signature scores were generated as described above. Pearson correlation coefficient r was calculated using R software. To test whether Yap signaling activation is an adaptive response to chemotherapy, gene expression data from two datasets, which included 25 and 48 matched breast cancer patient specimens collected before and after chemotherapy, were obtained from the Gene Expression Omnibus (GEO) website with accession numbers GSE21974 and GSE4382, respectively. A 57-gene *Yap1* signature found in breast cancer^19^ was adopted to calculate the *Yap* signature score as described above. To test whether increased Yap1-PTGS2 signaling in response to chemotherapy is associated with therapeutic resistance, *Yap1-PTGS2* signature scores of each breast cancer patient specimens collected before and after treatment (GSE21974) were generated as described previously. Change of the score after chemotherapy and the percentage of the remaining tumor volume were calculated for each patient.

All quantitative experiments were conducted using at least two independent biological repeats and are presented as mean ± SD. Quantitative data were analyzed by *t*-test unless otherwise mentioned. *P* < 0.05 (two-sided) was considered statistically significant.

## Supplementary information

Supplementary figure 1-15

## Data Availability

Further information and requests for resources and software should be addressed to D.Y. (mail to: dyu@mdanderson.org).
