# Supplementary material for: Targeting a chemo-induced adaptive signaling circuit confers therapeutic vulnerabilities in pancreatic cancer
Source: Cell Discov. 2024 Oct 29;10:109. doi: 10.1038/s41421-024-00720-w (PMC11519973; doi:10.1038/s41421-024-00720-w)
Supplement: Supplementary file 1 — Supplementary figure 1-15 [file 41421_2024_720_MOESM1_ESM.pdf]

Supplementary information, Fig. S1

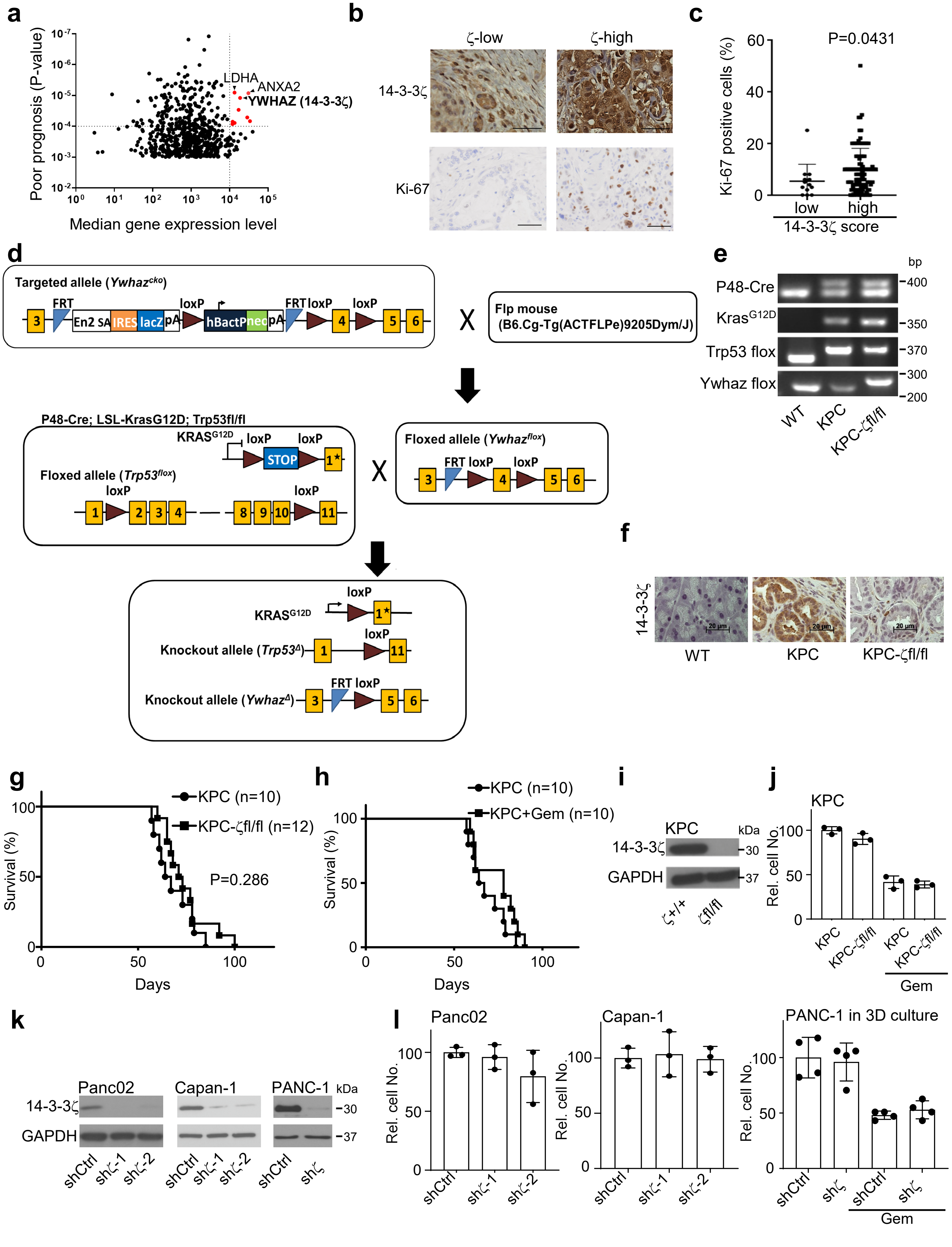

Supplementary information, Fig. S1. 14-3-3ζ is a prognostic factor for poor outcomes human PDAC and murine PDAC treated with gemcitabine.

a, Dot plot of gene expression levels and prognostic *P* value of 668 genes associated with poor prognosis in the TCGA PDAC patient dataset generated using the method described at https://www.proteinatlas.org/humanpathology/pancreatic+cancer. **b,** Representative IHC images of PDACs from MDACC PDAC patient cohort 1 stained with 14-3-3ζ and Ki-67. 14-3-3ζ expression levels were evaluated (score 1-3) by three pathologists independently. Score 1: 14-3-3ζ-low, score 2-3: 14-3-3ζ-high. Scale bar: 20μm (14-3-3ζ), 50μm (Ki-67). **c,** Comparison of Ki-67-positive tumor cells (%) in 14-3-3ζ-low versus -high expressing PDACs (mean ± SD, Mann-Whitney test). **d,** Diagram showing the breeding strategies to generate pancreas-specific 14-3-3ζ conditional knockout mice. **e,** Genotyping analysis of P48-Cre, $Kras^{G12D}$, $Trp53^{flox}$, and Ywhaz $(14\text{-}3\text{-}3\zeta)^{flox}$ in C57BL/6 WT mice, KPC mice, and KPC-$\zeta^{fl/fl}$ mice. **f,** Representative IHC images of pancreas tissue from WT C57BL/6 mice and PDAC tumor tissues from KPC and KPC-$\zeta^{fl/fl}$ mice stained with 14-3-3ζ. Representative image of three independent repeats. **g,** Kaplan-Meier survival analysis of KPC (n = 10) and KPC-$\zeta^{fl/fl}$ (n = 12) mice (log-rank test). **h,** Kaplan-Meier survival analysis of KPC mice treated with/without Gem. (Log-Rank test). The survival curves of KPC mice without Gem-treatment were adopted from Supplementary Fig. 1g, and survival curves of KPC mice with Gem-treatment were adopted from Fig. 1b. **i,** WB analysis of 14-3-3ζ and GAPDH (sample processing controls) expression in PDAC cells established from KPC and KPC-$\zeta^{fl/fl}$ mice. Representative data of two independent repeats. **j,** Relative cell numbers of KPC and KPC-$\zeta^{fl/fl}$ cells treated with or without Gem (8.5 nM) for 48 h (mean ± SD, t-test, n = 3 biological repeats). **k,** WB analysis of 14-3-3ζ and GAPDH (sample processing controls) expression in PDAC.shCtrl and PDAC.shζ cell lines (Panc02, Capan-1, PANC-1). Representative data of two independent repeats. **l,** Relative cell numbers of PDAC.shCtrl and PDAC.shζ cells treated with vehicle/Gem (20 nM) for 48 h (Panc02) and for 72 h (Capan-1, 3D-cultured PANC-1). (Mean ± SD, t-test, n = 3-4 biological repeats).

Supplementary information, Fig. S2

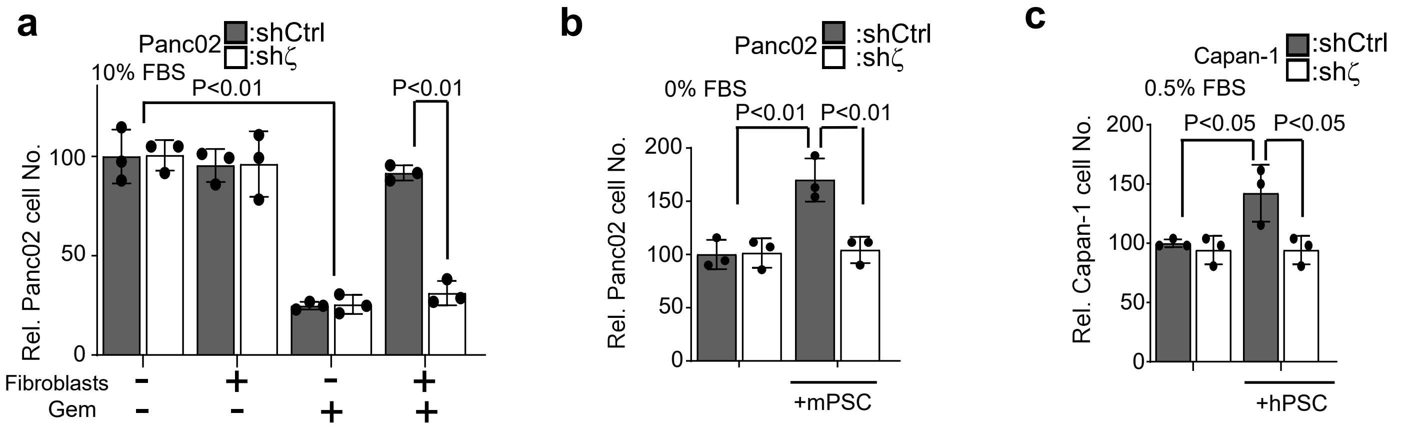

Supplementary information, Fig. S2. 14-3-3ζ-overexpressing PDAC enlists PSCs/fibroblasts to confer stress resistance.

a, Relative cell number of Panc02.shCtrl/shζ cells co-cultured with or without NIH3T3 cells under Gem treatment (8.5 nM) for 48 h. Panc02 cell:NIH3T3 cell =1:9. (Mean ± SD, *t*-test, n = 3 biological repeats). **b,** Relative cell number of Panc02.shCtrl/shζ cells co-cultured with or without mPSCs in 0% FBS medium for 48 h. (Panc02 cell:mPSC = 1:1. Mean ± SD, *t*-test, n = 3 biological repeats). **c,** Relative cell number of Capan-1.shCtrl/shζ cells co-cultured with or without hPSCs in 0.5% FBS medium for 72 h. Capan-1 cell:hPSC = 1:1. (Mean ± SD, *t*-test, n = 3 biological repeats).

Supplementary information, Fig. S3

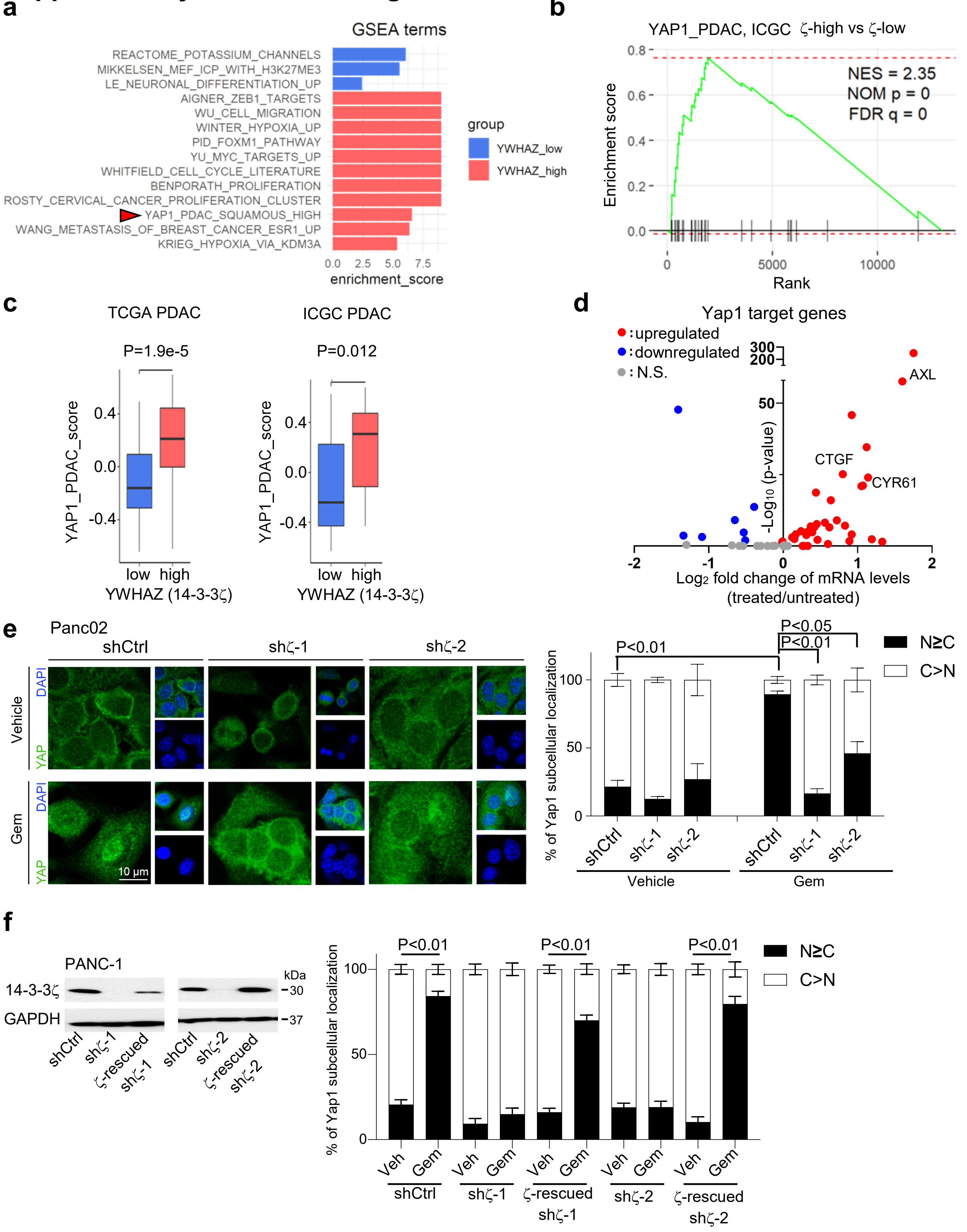

Supplementary information, Fig. S3. Stresses induce Yap1 activation in 14-3-3ζ overexpressing PDAC cells.

a, Gene Set Enrichment (GSE) analysis of 14-3-3ζ-high and -low PDACs in the TCGA PDAC patient dataset. **b,** The enrichment score of Yap1 target gene signature in 14-3-3ζ high/low PDAC in the ICGC PDAC patient dataset. **c,** Comparison of the Yap1 target gene signature scores in 14-3-3ζ-high and -low PDACs in TCGA (left) and ICGC (right) patient datasets. **d,** Single-nucleus RNA-sequence analysis of Yap1 target gene expression in chemoradiotherapy-treated vs. untreated PDAC tumors (data adopted from ref.[18] of human PDAC patient dataset). **e,** IF staining of Yap1 in Panc02.shCtrl and Panc02.shζ cells with or without Gem (20 nM) for 3 h (mean ± SEM, two-way ANOVA, 10 representative pictures for each group). **f,** Left: WB analyses of 14-3-3ζ and GAPDH (sample processing controls) in PACN-1.shCtrl, PACN-1.shζ, and 14-3-3ζ overexpressing PACN-1.shζ cells. Right: IF staining of Yap1 in PANC-1.shCtrl, PANC-1.shζ, and 14-3-3ζ overexpressing PACN-1.shζ cells with or without Gem (20 nM) for 3 h (mean ± SEM, two-way ANOVA, 10 representative pictures for each group).

Supplementary information, Fig. S4

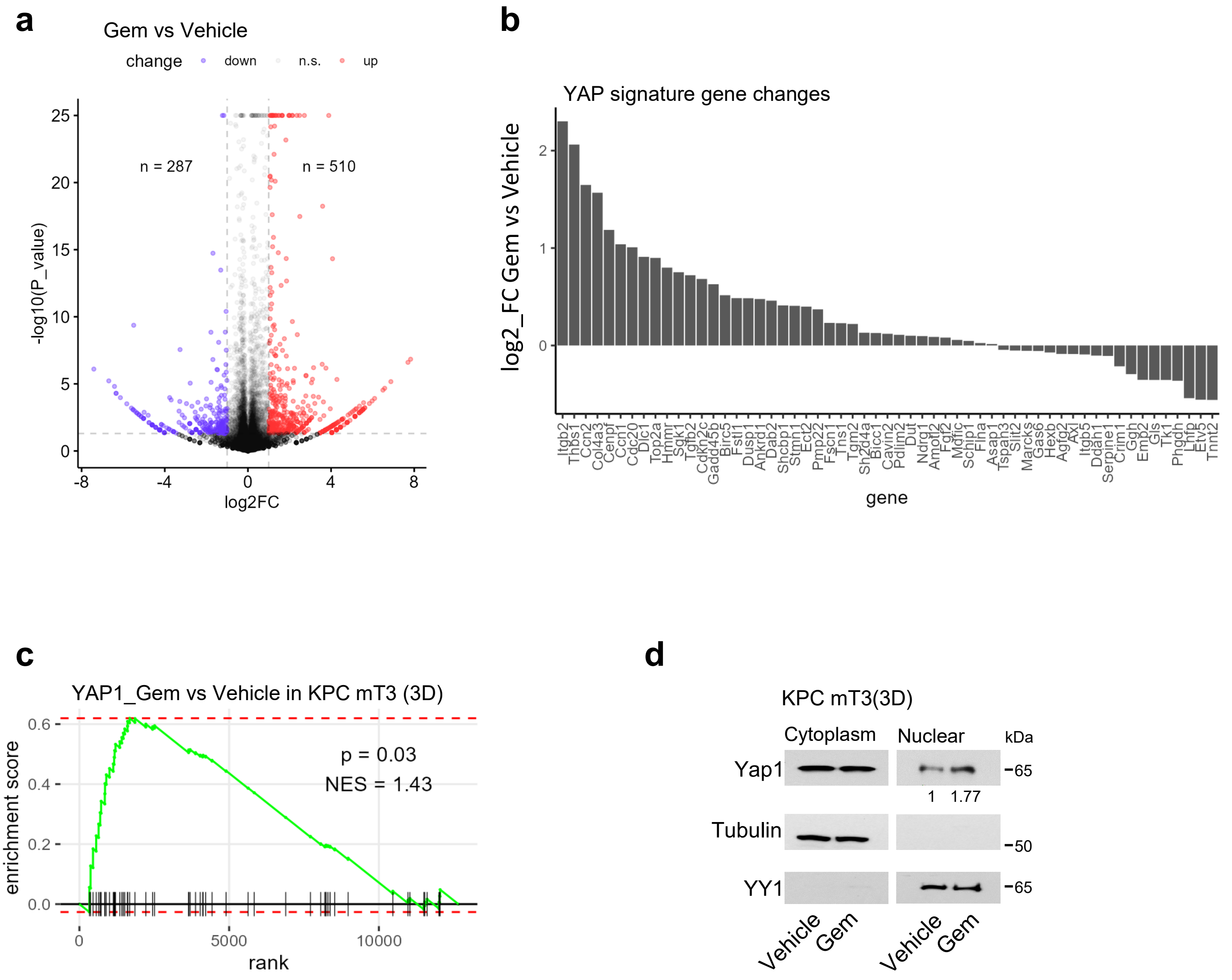

Supplementary information, Fig. S4. RNA-seq analysis of Yap1 target genes in KPC mT3 cells treated with vehicle or gemcitabine.

a, Volcano plot of differential gene expression between Vehicle-treated and Gem (20 nM, 72 h)-treated KPC mT3 cells growing in 3D culture. **b,** Fold changes of Yap1 core downstream gene (57 genes) expression in Gem (20 nM, 72 h)-treated 3D-cultured KPC mT3 cells compared to Vehicle treated 3D-cultured KPC mT3 cells. **c,** Gene set enrichment analysis of Yap1 signature in 3D-cultured KPC mT3 cells treated with Gem (20 nM)/vehicle for 72 h. **d,** WB analyses of cytoplasmic and nuclear Yap1, tubulin (a cytoplasmic protein marker, sample processing controls), and YY1 (a nuclear protein marker, sample processing controls) in 3D-cultured KPC mT3 cells treated with Gem (20 nM) or vehicle for 1 h. Representative data of two independent repeats.

Supplementary information, Fig. S5

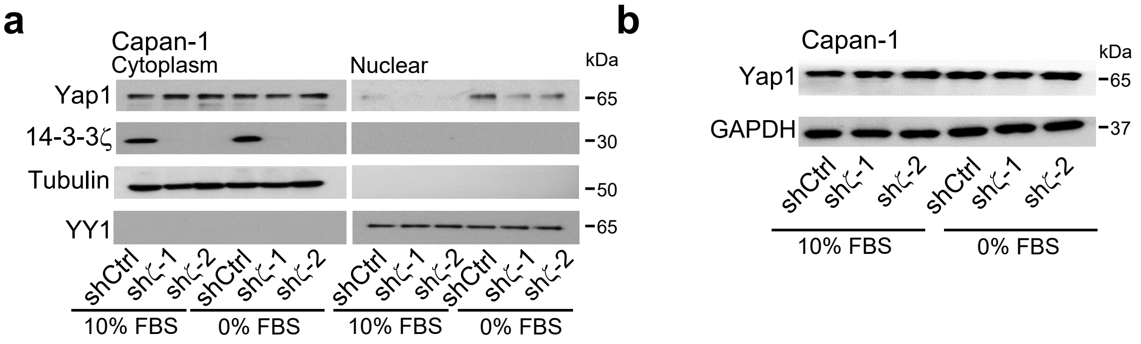

Supplementary information, Fig. S5. Low-nutrient induces Yap1 nuclear accumulation in 14-3-3ζ+++ cancer cells.

a, WB analysis of nuclear and cytoplasmic Yap1, 14-3-3ζ, tubulin (sample processing controls), and YY1 (sample processing controls) in Capan-1.shCtrl and Capan-1.shζ cells cultured in 10% or 0% FBS medium for 72 h. Representative data of two independent repeats. **b,** WB analysis of Yap1 and GAPDH (sample processing controls) in Capan-1.shCtrl cells and Capan-1.shζ cells in 10% or 0% FBS medium for 72 h. Representative data of two independent repeats.

Supplementary information, Fig. S6

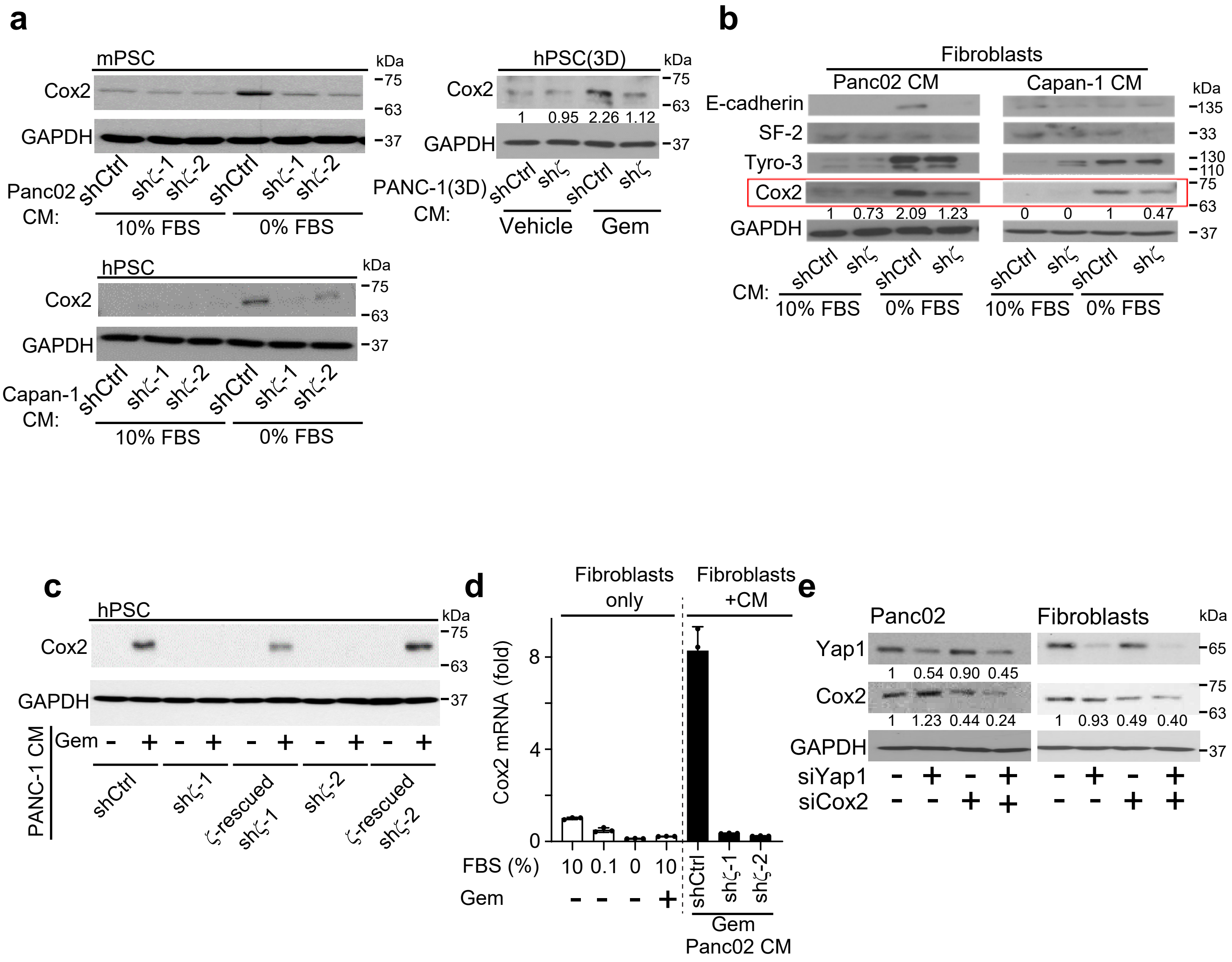

Supplementary information, Fig. S6. Stressed 14-3-3 ζ+++ cancer cells induce Cox2 expression in PSCs/fibroblasts.

a, Left: WB analysis of Cox2 and GAPDH (as sample processing controls) in mPSCs or hPSCs treated with CM from Panc02.shCtrl/shζ (for mPSCs) or Capan1.shCtrl/shζ (for hPSCs) cells that were cultured in 10% or 0% FBS. Right: WB analysis of Cox2 and GAPDH (sample processing controls) in 3D-cultured hPSCs treated with CM from 3D-cultured PANC-1.shCtrl or shζ cells treated with or without Gem (8.5 nM, 72 h). Representative data of two independent repeats. **b,** WB analysis of E-cadherin, SF-2, Tyro-3, Cox2, and GAPDH (sample processing controls) in NIH3T3 cells treated for 24 h with CM from either Panc02.shCtrl/shζ cells or Capan-1.shCtrl/shζ cells that were cultured in 10% or 0% FBS for 72 h. **c,** WB analysis of Cox2 and GAPDH (as sample processing controls) in hPSCs treated with CM from PANC-1.shCtrl, shζ, or 14-3-3ζ overexpressing shζ cells treated with or without Gem (20nM, 72h). Representative data of two independent repeats. **d,** qRT-PCR of Cox2 mRNA expression in NIH3T3 cells incubated for 5 h with vehicle/Gem (8.5 nM) in 10% FBS/0.1% FBS medium, or CM collected from Panc02.shCtrl or shζ cells treated with Gem (8.5 nM) for 48 h (mean ± SD, n = 3 biological repeats). All data are representative of at least two independent repeats. **e,** WB analysis of Yap1, Cox2, and GAPDH (sample processing controls) proteins in Panc02 cells and NIH3T3 cells transfected with Control siRNA or the indicated siRNAs. Representative data of two independent repeats.

Supplementary information, Fig. S7

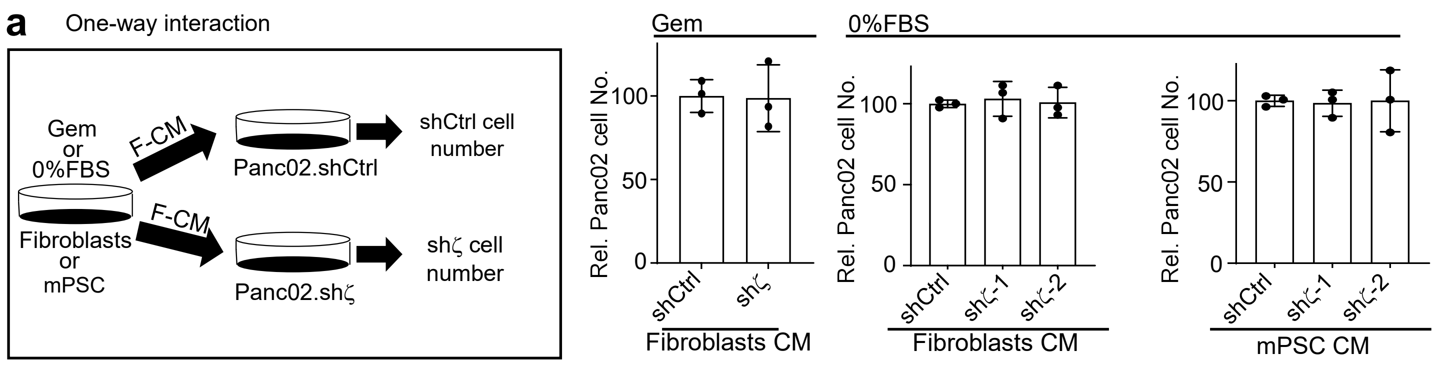

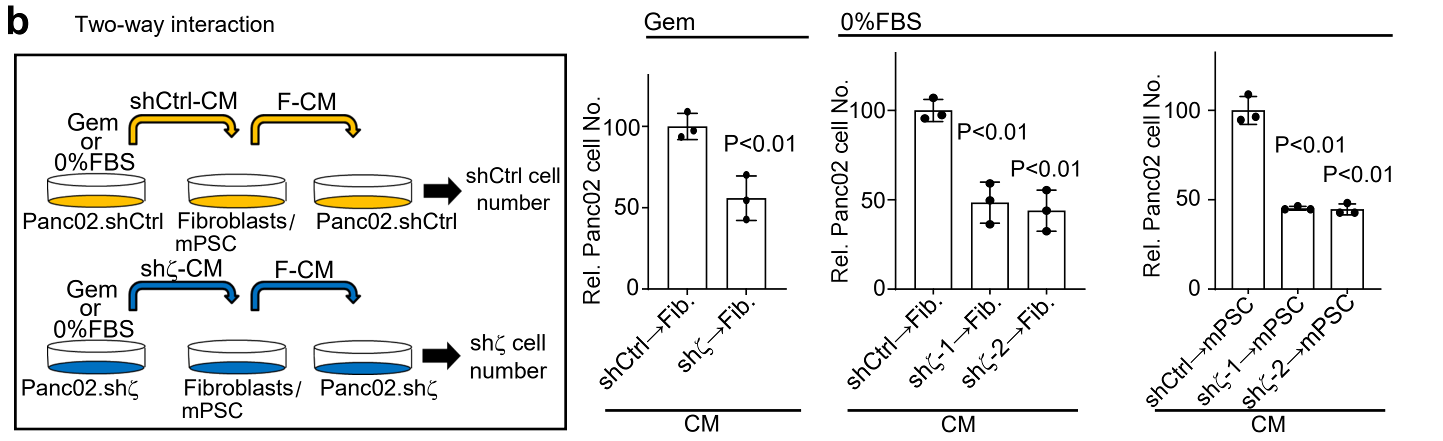

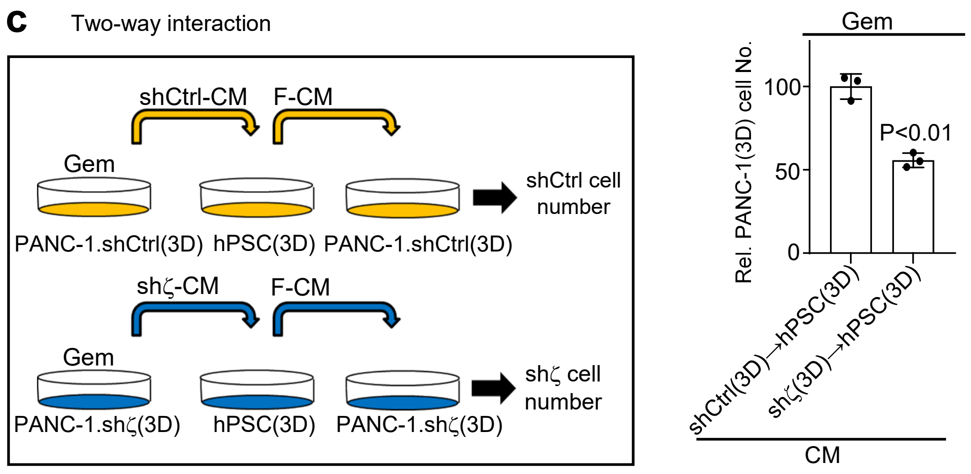

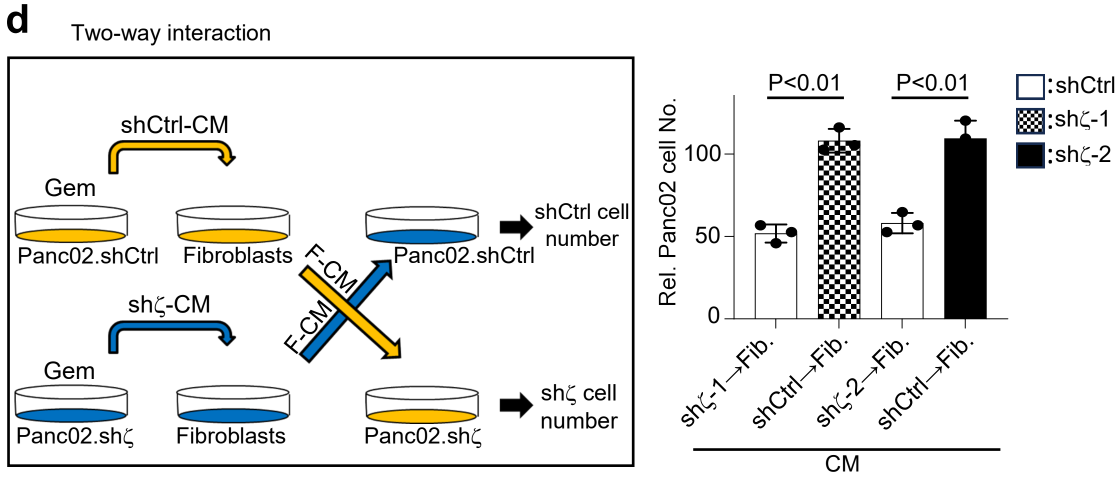

Supplementary information, Fig. S7. Symbiotic cooperation between 14-3-3ζ+++ PDAC cells and PSCs/fibroblasts facilitates PDAC growth and Gemcitabine resistance.

a, Relative cell numbers of Panc02.shCtrl and Panc02.shζ cells treated with CM collected from NIH3T3 cells or mPSC cultured in 0% FBS medium or treated with Gem in 10% FBS medium. Left: Schematic description of experiment (mean ± SD, *t*-test, n = 3 biological repeats). **b,** Relative cell numbers of Panc02.shCtrl and Panc02.shζ cells treated with F-CM collected from NIH3T3 cells or mPSCs activated by matched CM from Panc02.shCtrl or Panc02.shζ cells cultured in 0% FBS medium or treated with Gem in 10% FBS medium. Left: Schematic description of experiment (mean ± SD, *t*-test, n = 3 biological repeats). **c,** Relative cell numbers of 3D-cultured PANC-1.shCtrl and PANC-1.shζ cells treated with F-CM collected from 3D-cultured hPSCs activated by matched CM from 3D-cultured PANC-1.shCtrl cells/shζ cells treated with Gem (20 nM) in 10% FBS medium. Left: Schematic description of experiment (mean ± SD, *t*-test, n = 3 biological repeats). **d,** Relative cell numbers of Panc02.shCtrl or Panc02.shζ cells treated with F-CM collected from NIH3T3 cells activated by CM from Panc02.shζ or shCtrl cells, respectively. PDAC CM was collected from Panc02.shCtrl or shζ cells treated with Gem in 10% FBS medium. Left: Schematic description of experiment (mean ± SD, *t*-test, n = 3 biological repeats).

Supplementary information, Fig. S8

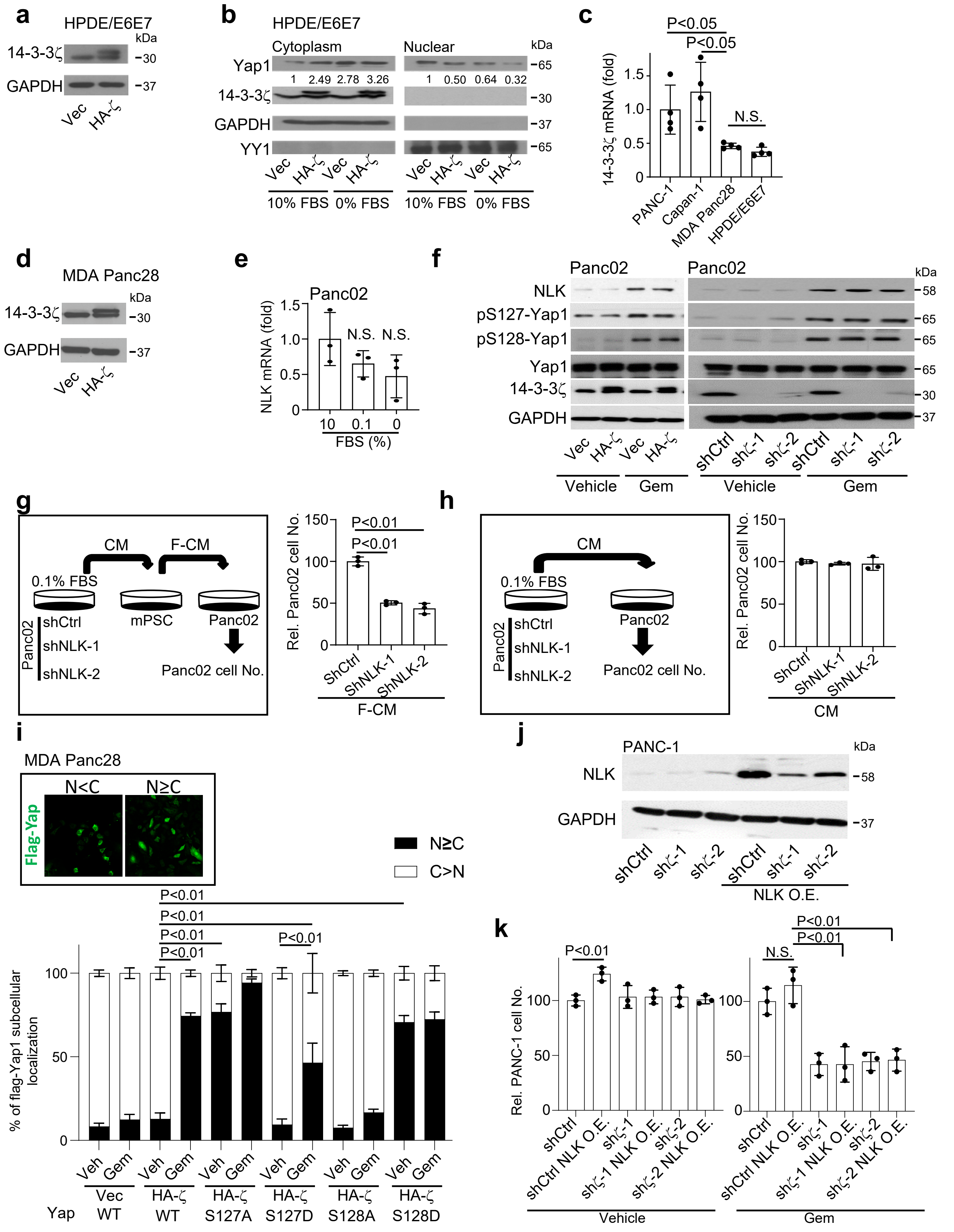

Supplementary information, Fig. S8. Stress induces Yap1 nuclear accumulation in 14-3-3ζ+++ PDAC cells but not in non-transformed pancreatic epithelial cells.

a, WB analysis of 14-3-3ζ and GAPDH (sample processing controls) expression in HPDE/E6E7.vec and HPDE/E6E7.HA-ζ cells. Representative data of two independent repeats. **b,** WB analysis of nuclear and cytoplasmic Yap1, 14-3-3ζ, GAPDH (sample processing controls), and YY1 (sample processing controls) in HPDE/E6E7.vec cells and HPDE/E6E7.HA-ζ cells cultured in 10% or 0% FBS medium for 72 h. Representative data of two independent repeats. **c,** qRT-PCR analysis of 14-3-3ζ mRNA expression in PDAC cell lines and a non-transformed pancreatic epithelial cell line (HPDE/E6E7). (Mean ± SD, *t*-test, n = 4 biological repeats). **d,** WB analysis of 14-3-3ζ and GAPDH (sample processing controls) expression in MDA Panc28.vec cells and MDA Panc28.HA-ζ cells. Representative data of two independent repeats. **e,** qRT-PCR analysis of NLK mRNA expression in Panc02 cells cultured in 10%, 0.1%, or 0% FBS medium for 30 min (mean ± SD, *t*-test, n = 3 biological repeats). **f,** WB analysis of NLK, pS127-Yap1, pS128-Yap1, Yap1, 14-3-3ζ, and GAPDH (sample processing controls) in Panc02.Vec or HA-ζ (Left) or Panc02.shCtrl or shζ cells treated with Gem (20 nM) or vehicle for 30 min. Representative data of two independent repeats. **g,** Schematics and relative numbers of proliferating Panc02 cells treated with CM from mPSCs that were activated by CM from Panc02.shCtrl or Panc02.shNLK sublines cultured in 0.1% FBS for 48 h (mean ± SD, t-test, n = 3 biological repeats). **h,** Schematics and relative numbers of proliferating Panc02 cells treated with CM from Panc02.shCtrl or Panc02.shNLK sublines cultured in 0.1% FBS for 48 h (mean ± SD, t-test, n = 3 biological repeats). **i,** IF staining of Flag (Yap1 WT, S127A, S128A, S127D, and S128D mutants) in MDA Panc28.HA-ζ.Flag-Yap1 WT, S127A, S128A, S127D, or S128D cells treated with Gem-treatment (20 nM, 3 h) in 10% FBS medium (mean ± SEM, two-way ANOVA, 10 representative pictures for each group). **j,** WB analysis of NLK and GAPDH (sample processing control) in NLK-overexpressing/Vector PANC-1.shCtrl or shζ cells. Representative data of two independent repeats. **k,** Relative cell numbers of PANC-1 cells treated with F-CM collected from hPSCs activated by CM from NLK-overexpressing or vector PANC-1.shCtrl/shζ cells treated with/without Gem in 10% FBS medium. (mean ± SD, *t*-test, n = 3 biological repeats).

Supplementary information, Fig. S9

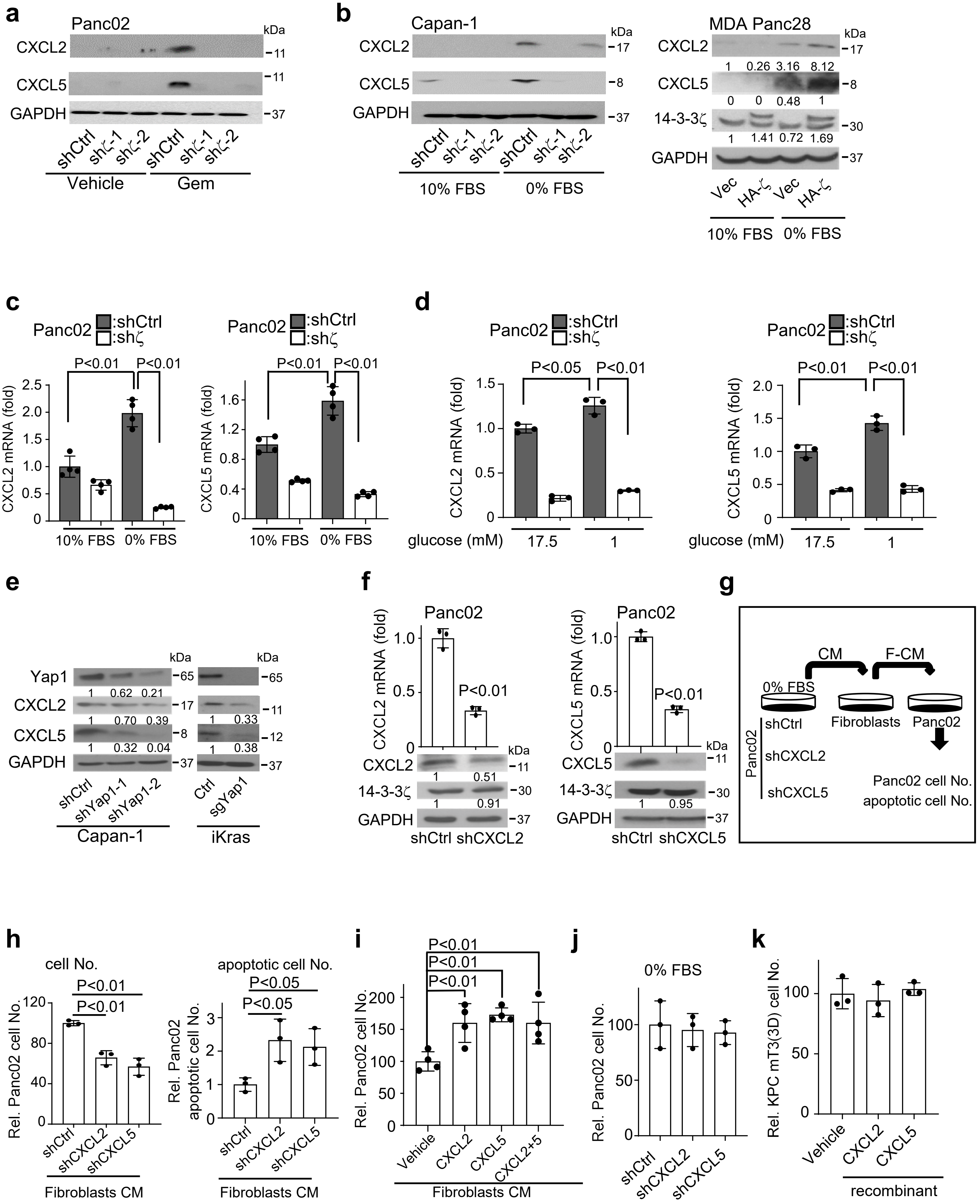

Supplementary information, Fig. S9. Stress induces CXCL2/5 via Yap1 in 14-3-3ζ+++ PDAC cells.

a, WB analysis of CXCL2, CXCL5, and GAPDH (sample processing controls) in Panc02.shCtrl and Panc02.shζ cells treated with Gem (20 nM) or vehicle for 48 h. **b,** WB analysis of CXCL2, CXCL5, and GAPDH (sample processing controls) protein expression in Capan-1.shCtrl and Capan-1.shζ cells cultured in 10% or 0% FBS medium for 72 h (Left), or in MDA Panc28.Vec and MDA Panc28.HA-ζ cells cultured in 10% or 0% FBS medium for 48 h (Right). **c,** qRT-PCR analysis of CXCL2 and CXCL5 mRNA expression in Panc02.shCtrl and Panc02.shζ cells cultured in 10% or 0% FBS medium for 72 h (mean ± SD, *t*-test, n = 4 biological repeats). **d,** qRT-PCR analysis of CXCL2 and CXCL5 mRNA expression in Panc02.shCtrl and Panc02.shζ cells cultured in 17.5 mM or 1 mM glucose medium for 6 h (CXCL2) or 24 h (CXCL5) (mean ± SD, *t*-test, n = 3 biological repeats). **e,** WB analyses of Yap1, CXCL2, CXCL5, and GAPDH (sample processing controls) protein expression in Capan-1.shCtrl/shYap1 cells (Left) and iKras.Ctrl/sgYap1 cells (Right) cultured in 0% FBS medium for 72 h (Capan-1cells) or for 48 h (iKras cells). **f,** qRT-PCR analysis of CXCL2/5 expression and WB analysis of CXCL2/5, 14-3-3ζ and GAPDH (sample processing controls) protein expression in Panc02.shCtrl, Panc02.shCXCL2, and Panc02.shCXCL5 cells (mean ± SD, *t*-test, n = 3 biological repeats). **g-h,** Schematic description of the experiment (g) and relative cell numbers (h, Left) and apoptotic cell numbers (h, Right) of Panc02 cells treated with CM collected from NIH3T3 cells that were activated by CM from Panc02.shCtrl, Panc02.shCXCL2, or Panc02.shCXCL5 cells cultured in 0% FBS medium (mean ± SD, t-test, n = 3 biological repeats). **i,** Relative cell numbers of Panc02 cells treated with CM from NIH3T3 cells with vehicle, recombinant CXCL2 (0.5 ng/mL), or recombinant CXCL5 (0.1 µg/mL) for 48 h (mean ± SD, *t*-test, n = 4 biological repeats). **j,** Relative cell numbers of Panc02.shCtrl, Panc02.shCXCL2, or Panc02.shCXCL5 cells cultured in 0% FBS medium for 48 h (mean ± SD, t-test, n = 3 biological repeats). **k,** Relative cell numbers of 3D-cultured KPC mT3 cells treated with vehicle, recombinant CXCL2 (0.5 ng/mL), or recombinant CXCL5 (0.1 µg/mL) for 72 h (mean ± SD, *t*-test, n = 3 biological repeats). All data are representative of at least two independent repeats.

Supplementary information, Fig. S10

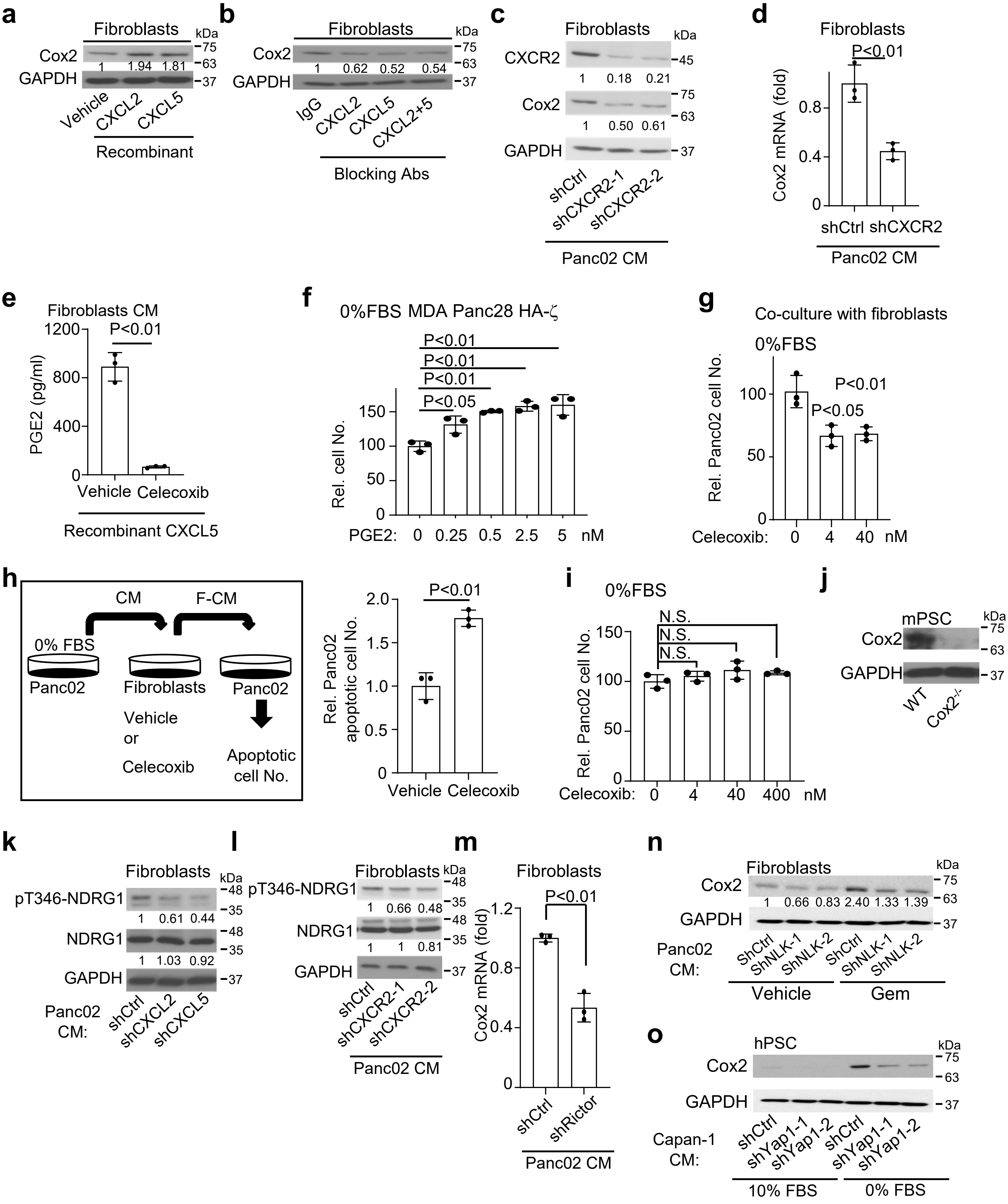

Supplementary information, Fig. S10. Stromal fibroblast CXCR2-mTORC2-Cox2-PGE2 axis fuels PDAC cell growth under stress.

a, WB analyses of Cox2 and GAPDH (sample processing controls) in NIH3T3 cells incubated with recombinant CXCL2 (0.5 ng/mL) or CXCL5 (0.1 μg/mL) for 8 h. **b,** WB analyses of Cox2 and GAPDH (sample processing controls) in NIH3T3 cells treated with CM from Panc02 (0% FBS, 72 h) cells added with IgG or CXCL2 (4 μg/mL) and CXCL5 (2.5 μg/mL) blocking antibodies for 24 h. **c,** WB analysis of CXCR2, Cox2, and GAPDH (sample processing controls) proteins in NIH3T3.shCtrl and NIH3T3.shCXCR2 cells treated with CM collected from Panc02 cells cultured in 0% FBS medium for 72 h. **d,** qRT-PCR analysis of Cox2 mRNA expression in NIH3T3.shCtrl and NIH3T3.shCXCR2 cells treated with CM from Panc02 cells cultured in 0% FBS for 72 h (mean ± SD, *t*-test, n = 3 biological repeats). **e,** PGE2 concentration in CM collected from NIH3T3 cells treated with recombinant CXCL5 (0.1 μg/mL) with or without Celecoxib (40 nM) for 48 h (mean ± SD, *t*-test, n = 3 biological repeats). **f,** Relative cell numbers of MDA Panc28.HA-ζ cells treated with PGE2 in 0% FBS for 48 h (mean ± SD, *t*-test, n = 3 biological repeats). **g,** Relative numbers of Panc02 cells co-cultured with NIH3T3 cells in the presence of Celecoxib in 0% FBS for 48 h (mean ± SD, *t*-test, n = 3 biological repeats). **h,** Relative apoptotic cell number of Panc02 cells treated for 48 h with CM collected from activated NIH3T3 cells treated with Celecoxib (40 nM) for 48 h. Left: Schematic description of experiment (mean ± SD, *t*-test, n = 3 biological repeats). **i,** Relative cell number of Panc02 cells treated with Celecoxib in 0% FBS for 48 h (mean ± SD, *t*-test, n = 3 biological repeats). **j,** WB analysis of Cox2 and GAPDH (sample processing controls) protein in WT and $Cox2^{-/-}$ mPSCs treated for 24 h with CM collected from Panc02 cells cultured in 0% FBS medium for 48 h. **k,** WB analysis of pT346-NDRG1, NDRG1, and GAPDH (sample processing controls) expression in NIH3T3 cells treated for 12 h with CM collected from Panc02.shCtrl, Panc02.shCXCL2, or Panc02.shCXCL5 cells cultured in 0% FBS medium for 72 h. **l,** WB analysis of pT346-NDRG1, NDRG1, and GAPDH (sample processing controls) expression in NIH3T3.shCtrl and NIH3T3.shCXCR2 cells treated with CM collected from Panc02 cells cultured in 0% FBS medium for 72 h. **m,** qRT-PCR analysis of Cox2 mRNA expression in NIH3T3.shCtrl and NIH3T3.shRictor cells treated for 4 h with CM from Panc02 cultured in 0% FBS for 72 h (mean ± SD, *t*-test, n = 3 biological repeats). **n,** WB analysis of Cox2 and GAPDH (sample processing controls) protein in NIH3T3 cells treated with CM collected from Panc02.shCtrl or shNLK cells treated with Gem (20 nM, 72 h). All data are representative of at least two independent repeats. **o,** WB analysis of Cox2 and GAPDH (sample processing controls) protein in hPSCs treated with CM collected from Capan-1.shCtrl or shYap1 cells cultured in 0% FBS medium for 72 h. All data are representative of at least two independent repeats.

Supplementary information, Fig. S11

a

Late stage Panc02 orthotopic mouse model

	Vehicle	Gem	PPIX	Cel	PPIX Cel	Gem PPIX	Gem Cel	Gem PPIX Cel
Median survival after treatment (days)	18	19.5	17.5	18	16.5	33	17.5	57
P value (vs Gem)	0.77		0.88	0.46	0.71	0.003	0.84	0.00001

b

	Vehicle	Gem	Gem PPIX Cel
BUN (mg/dL)	36.6±16.8	31.9±14.1	24.3±2.1
P-value (vs Vehicle)		0.68	0.20
AST (U/L)	342.0±169.0	283.3±147.2	192.0±53.6
P-value (vs Vehicle)		0.62	0.14
ALT (U/L)	83.8±18.4	67.3±56.1	94.8±37.6
P-value (vs Vehicle)		0.60	0.62

c

Late stage KPC mouse model

	Vehicle	Gem	Gem PPIX	Gem Cel	Gem PPIX Cel
Median survival after treatment (days)	12	27	36	20.5	81.5
P value (vs Gem)	0.06		0.003	0.37	0.000004

d

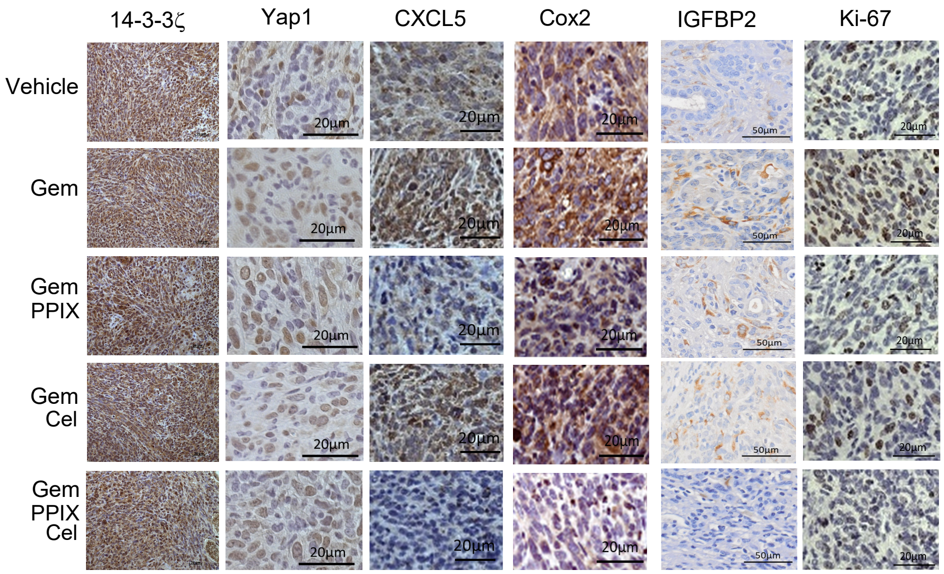

e **f** **g** **h** **i**

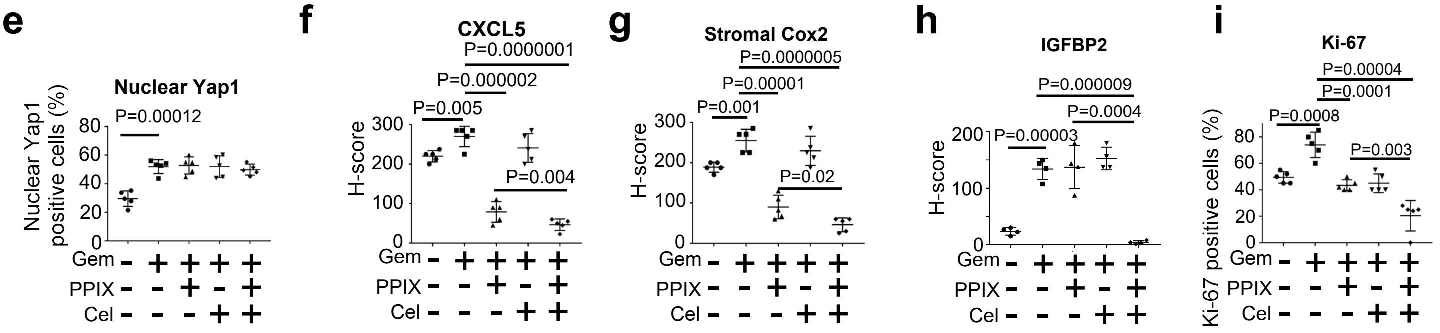

Supplementary information, Fig. S11. Assessment of the efficacy of co-targeting Yap1 and Cox2.

a, Summary of median survival analysis for late-stage Panc02 orthotopic mouse model treated with the indicated drugs. **b,** Assessment of the concentration of blood urea nitrogen (BUN), Aspartate transaminase (AST), and Alanine transaminase (ALT) in blood samples collected from mice bearing orthotopic Panc02 late-stage tumors treated with the indicated drugs for 1 week. **c,** Summary of median survival analysis for late-stage KPC mouse model treated with the indicated drugs. **d-i,** IHC staining of indicated proteins (horizontal labels) of Panc02 PDACs with indicated treatments (vertical labels) collected in experiments described in Fig. 6b (mean ± SD, *t*-test, n = 4-5 biological repeats).

Supplementary information, Fig. S12

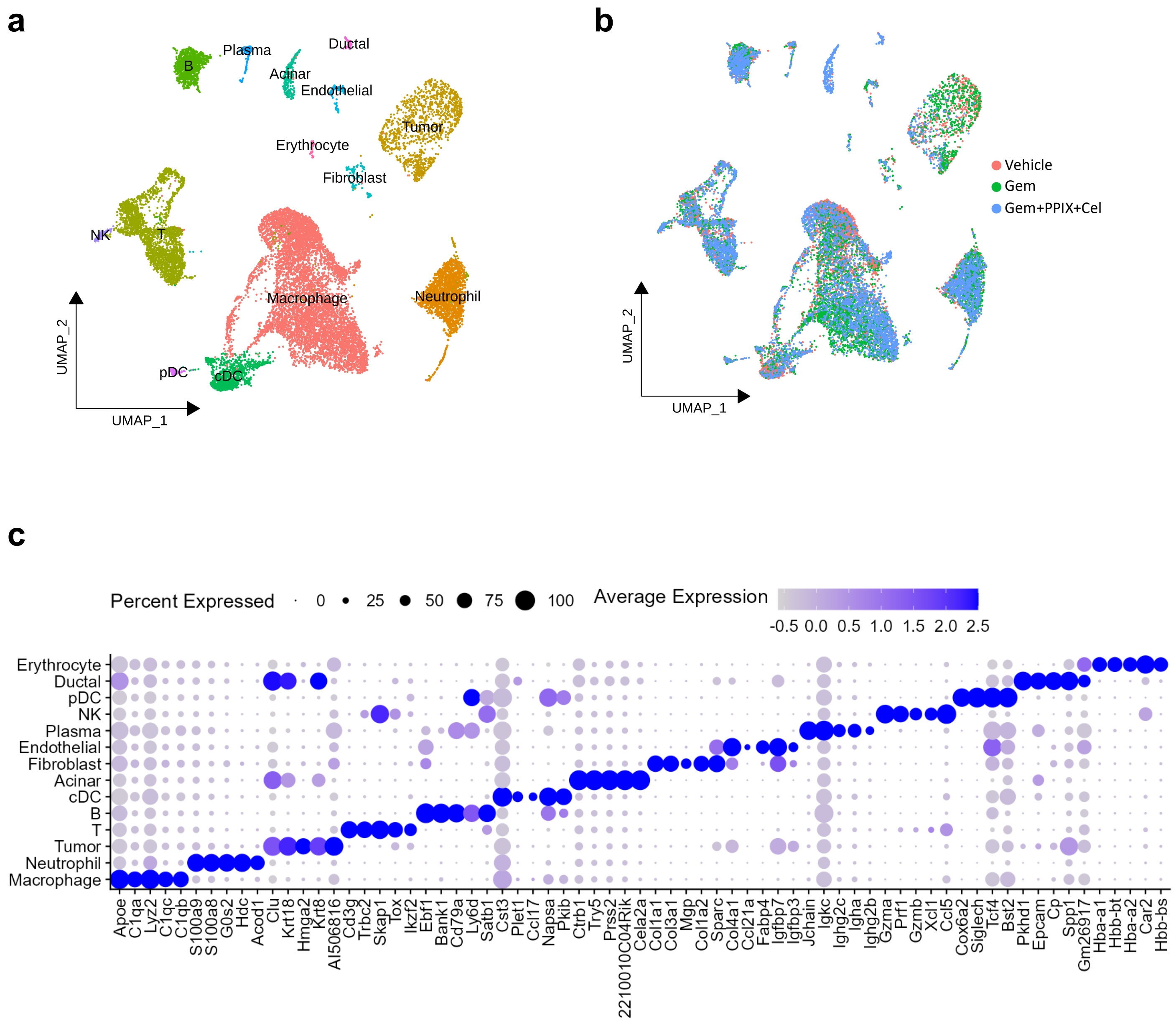

Supplementary information, Fig. S12. Profiling of immune populations in mouse PDACs.

a,b, UMAP visualization of the clustering of 16445 cells from vehicle-treated KPC mT3 tumor samples, Gem-treated samples, and co-targeting therapy-treated samples (Gem+PPIX+Cel), Color coded by major cell types (**a**) or treatment groups (**b**). **c,** Dotplot showing the expression of the top 5 most variable genes across each major cell type by scRNA-seq analysis of KPC mT3 tumor samples.

Supplementary information, Fig. S13

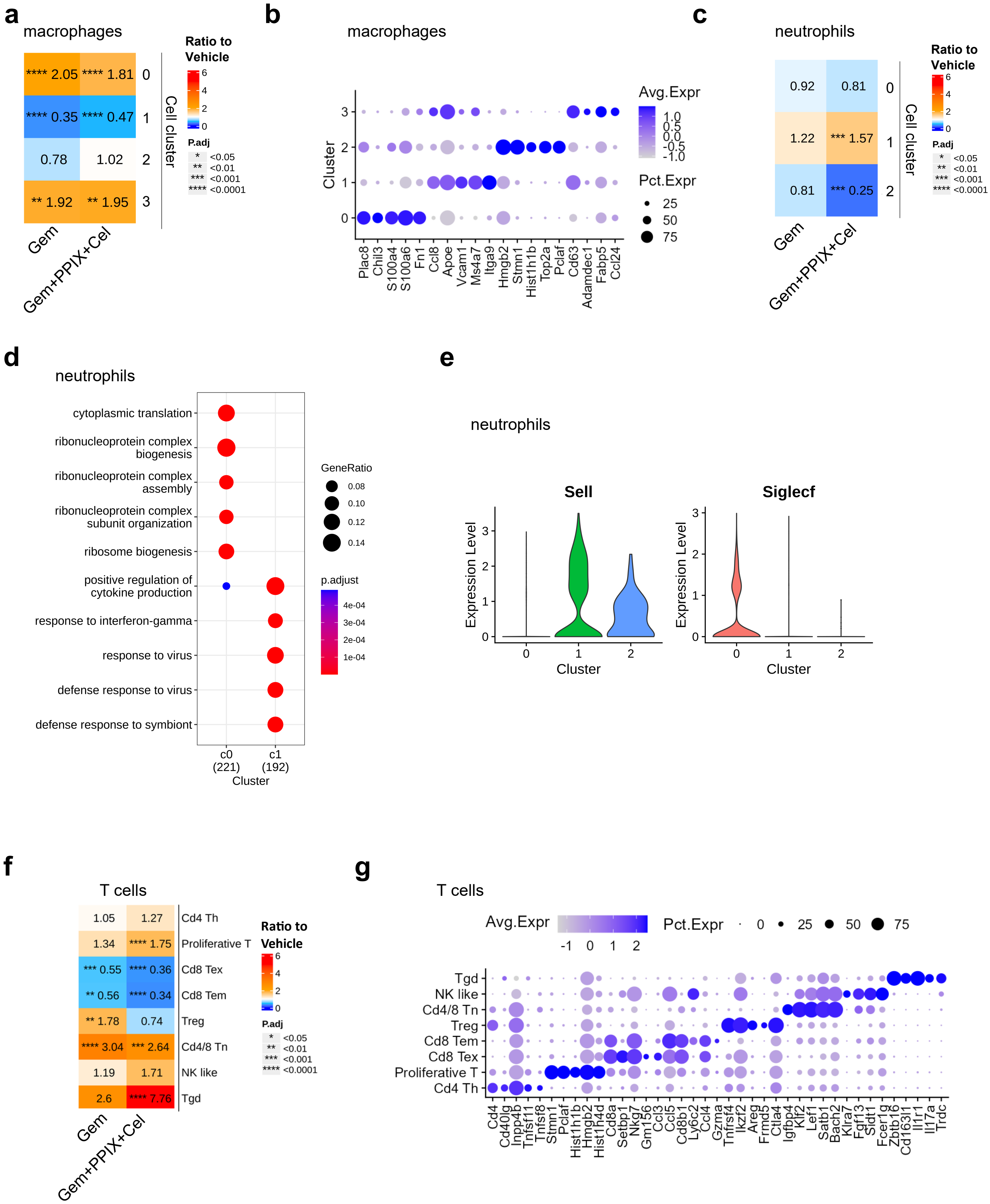

Supplementary information, Fig. S13. Co-targeting treatment reshapes the PDAC tumor immune microenvironment.

a, Relative frequencies of each macrophage (sub)cluster in Gem-treated KPC mT3 tumor samples and Gem+PPIX+Cel treated KPC mT3 tumor samples compared to vehicle-treated KPC mT3 tumor samples. The numbers indicate the ratio changes relative to the vehicle control samples. **b,** Dotplot showing the expression of 5 most variable genes across each macrophage (sub)cluster. **c,** Relative frequencies of each neutrophil (sub) cluster in Gem-treated KPC mT3 tumor samples and Gem+PPIX+Cel treated KPC mT3 tumor samples compared to vehicle-treated KPC mT3 tumor samples. The numbers indicate the ratio changes relative to the vehicle control samples. **d,** Enrichment of gene ontology (GO) terms of two major neutrophil (sub)clusters (C0 and C1). **e,** Expression of *Sell* and *Siglecf* in two neutrophil (sub)clusters. **f,** Relative frequencies of each T cell (sub) cluster in Gem-treated KPC mT3 tumor samples and Gem+PPIX+Cel treated KPC mT3 tumor samples compared to vehicle-treated KPC mT3 tumor samples. The numbers indicate the ratio changes relative to the vehicle-treated KPC mT3 tumor samples. **g,** Dotplot showing the expression of 5 most variable genes across each T cell (sub)cluster.

Supplementary information, Fig. S14

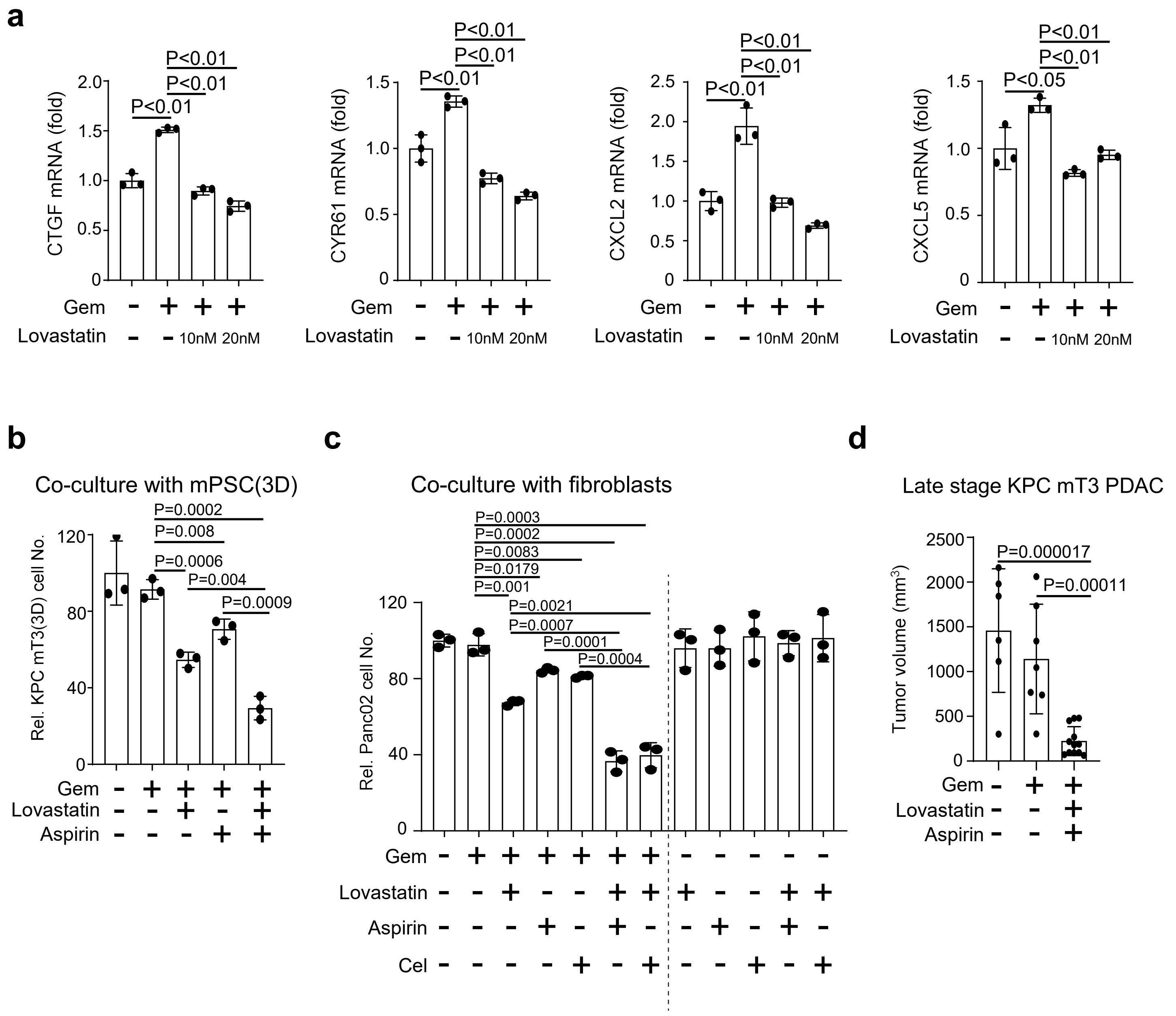

Supplementary information, Fig. S14. Co-targeting Yap1 and Cox2 by Lovastatin and Aspirin enhanced pancreatic cancer responses to Gem.

a, qRT-PCR analysis of CTGF, CYR61, CXCL2 and CXCL5 mRNA expression in Panc02 cells treated with Gem or Lovastatin (mean ± SD, *t*-test, n = 3 biological repeats). **b,** Relative cell number of KPC mT3 cells 3D-cultured in the lower chambers of a Transwell unit with 3D-cultured mPSCs treated with Gem (20 nM), Lovastatin (20 nM), Aspirin (10 µM), and/or Cel (4 nM) for 72 h (Mean ± SD, *t*-test, n = 3 biological repeats). **c,** Relative cell number of Panc02 cells co-cultured with NIH3T3 fibroblasts treated with Gem (8.5 nM), Lovastatin (20 nM), Aspirin (10 µM), Cel (4 nM), or in combination for 48 h (Mean ± SD, t-test, n = 3 biological repeats). **d,** Comparison of tumor volumes for mice bearing late-stage KPC mT3 tumors (15 days after KPC mT3 injection) after receiving 7 days of the indicated treatments, n=6 (Vehicle), n=7 (Gem), and n=12 (Gem+Lovastatin+Aspirin) (Mean ± SD, *t*-test).

Supplementary information, Fig. S15

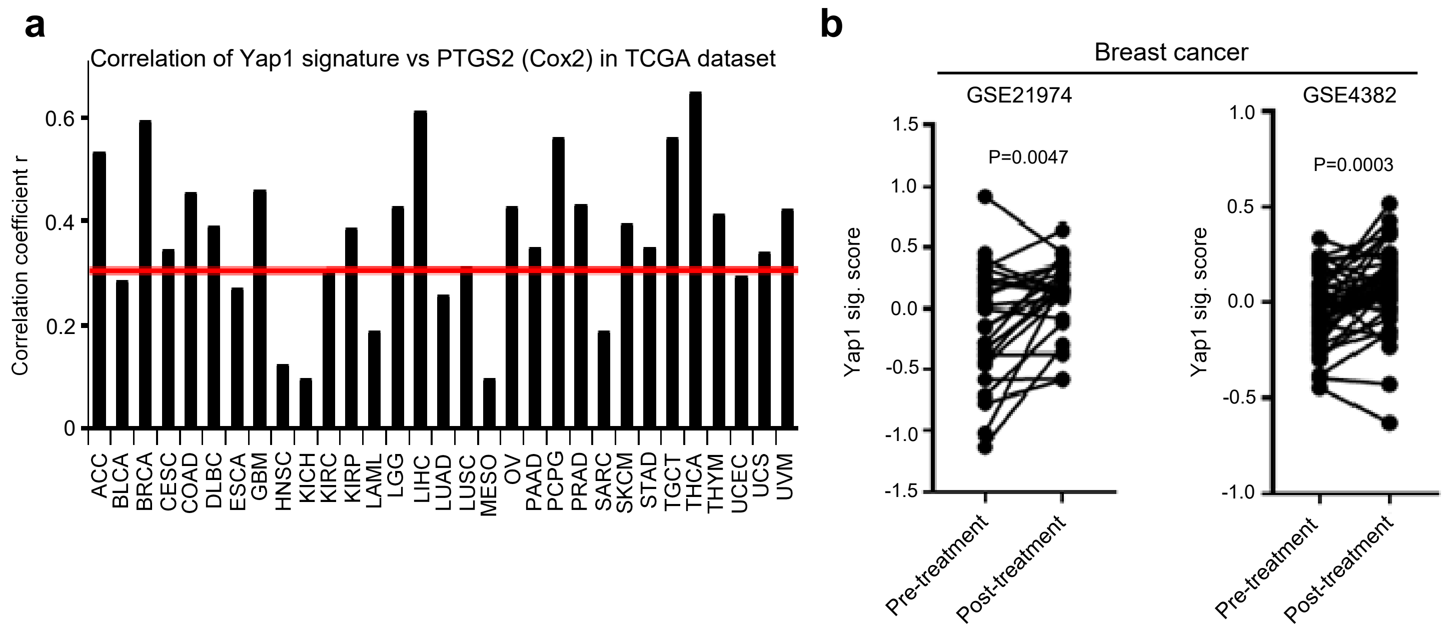

Supplementary information, Fig. S15. Adaptive upregulation of Yap1 and Cox2 contributes to chemotherapeutic resistance in other cancer types.

a, Analysis of Pearson correlation between a 57-gene YAP1 signature and PTGS2 (Cox2) mRNA expression in different cancer types from TCGA datasets. Red line indicates the cutoff of Pearson correlation coefficient r = 0.3. The cancer types include: adrenocortical carcinoma (ACC), bladder urothelial carcinoma (BLCA), breast invasive carcinoma (BRCA), cervical squamous cell carcinoma and endocervical adenocarcinoma (CESC), colon adenocarcinoma (COAD), lymphoid neoplasm diffuse large B-cell lymphoma (DLBC), esophageal carcinoma (ESCA), glioblastoma multiforme (GBM), head and neck squamous cell carcinoma (HNSC), kidney chromophobe (KICH), kidney renal clear cell carcinoma (KIRC), kidney renal papillary cell carcinoma (KIRP), acute myeloid leukemia (LAML), brain lower grade glioma (LGG), liver hepatocellular carcinoma (LIHC), lung adenocarcinoma (LUAD), lung squamous cell carcinoma (LUSC), mesothelioma (MESO), ovarian serous cystadenocarcinoma (OV), pancreatic adenocarcinoma (PAAD), pheochromocytoma and paraganglioma (PCPG), prostate adenocarcinoma (PRAD), sarcoma (SARC), skin cutaneous melanoma (SKCM), stomach adenocarcinoma (STAD), testicular germ cell tumors (TGCT), thyroid carcinoma (THCA), thymoma (THYM), uterine corpus endometrial carcinoma (UCEC), uterine carcinosarcoma (UCS), uveal melanoma (UVM). **b,** Analysis of Yap1 signature scores of matched breast cancer patient specimens collected before and after chemotherapy (GSE21974, n = 25, *pair-t* test; GSE4382, n = 48, *pair-t* test).
